# Shaping Embodied Neural Networks for Adaptive Goal-directed Behavior

**DOI:** 10.1371/journal.pcbi.1000042

**Published:** 2008-03-28

**Authors:** Zenas C. Chao, Douglas J. Bakkum, Steve M. Potter

**Affiliations:** Laboratory for Neuroengineering, Department of Biomedical Engineering, Georgia Institute of Technology and Emory University School of Medicine, Atlanta, Georgia, United States of America; University College London, United Kingdom

## Abstract

The acts of learning and memory are thought to emerge from the modifications of synaptic connections between neurons, as guided by sensory feedback during behavior. However, much is unknown about how such synaptic processes can sculpt and are sculpted by neuronal population dynamics and an interaction with the environment. Here, we embodied a simulated network, inspired by dissociated cortical neuronal cultures, with an artificial animal (an animat) through a sensory-motor loop consisting of structured stimuli, detailed activity metrics incorporating spatial information, and an adaptive training algorithm that takes advantage of spike timing dependent plasticity. By using our design, we demonstrated that the network was capable of learning associations between multiple sensory inputs and motor outputs, and the animat was able to adapt to a new sensory mapping to restore its goal behavior: move toward and stay within a user-defined area. We further showed that successful learning required proper selections of stimuli to encode sensory inputs and a variety of training stimuli with adaptive selection contingent on the animat's behavior. We also found that an individual network had the flexibility to achieve different multi-task goals, and the same goal behavior could be exhibited with different sets of network synaptic strengths. While lacking the characteristic layered structure of *in vivo* cortical tissue, the biologically inspired simulated networks could tune their activity in behaviorally relevant manners, demonstrating that leaky integrate-and-fire neural networks have an innate ability to process information. This closed-loop hybrid system is a useful tool to study the network properties intermediating synaptic plasticity and behavioral adaptation. The training algorithm provides a stepping stone towards designing future control systems, whether with artificial neural networks or biological animats themselves.

## Author Summary

The ability of a brain to learn has been studied at various levels. However, a large gap exists between behavioral studies of learning and memory and studies of cellular plasticity. In particular, much remains unknown about how cellular plasticity scales to affect network population dynamics. In previous studies, we have addressed this by growing mammalian brain cells in culture and creating a long-term, two-way interface between a cultured network and a robot or an artificial animal. Behavior and learning could now be observed in concert with the detailed and long-term electrophysiology. In this work, we used modeling/simulation of living cortical cultures to investigate the network's capability to learn goal-directed behavior. A biologically inspired simulated network was used to determine an effective closed-loop training algorithm, and the system successfully exhibited multi-task goal-directed adaptive behavior. The results suggest that even though lacking the characteristic layered structure of a brain, the network still could be functionally shaped and showed meaningful behavior. Knowledge gained from working with such closed-loop systems could influence the design of future artificial neural networks, more effective neuroprosthetics, and even the use of living networks themselves as a biologically based control system.

## Introduction

One of the most important features of the brain is the ability to adapt or learn to achieve a specific goal, which requires continuous sensory feedback about the success of its motor output in a specific context. We developed tools [1]–[3] for closing the sensory-motor loop between a cultured network and a robot or an artificial animal (animat) [4] in order to study learning directly through behavior of the artificial body and its interaction with its environment. Compared to animal models, the cultured network is a simpler and more controllable system to investigate basic network computations; confounding factors such as sensory inputs, attention, and behavioral drives are absent, while diverse and complex activity patterns remain [5]–[9].

Previously, an embodied cultured network's ability to control an animat or a mobile robot was demonstrated without a specifically defined goal [2],[10]. In another case, animats were designed to avoid obstacles [11] or follow objects [12], but deterministically and without learning. By using a lamprey brainstem to control a mobile robot, Mussa-Ivaldi et al. demonstrated the embodied *in vitro* network's tendency to compensate the sensory imbalance caused by artificially altering the sensitivity of the sensors at one side of the robot. Without a pre-defined goal and external training stimulation, long-term changes in behavior in response to the sensory imbalance were found in embodied lamprey brainstems [13], however, the changes were unpredictable [14]. In order to further understand the learning capability of an embodied cultured network for goal-directed behavior, we need to investigate how the network can be shaped and rewired, and how to direct this change.

Previous studies have demonstrated the potential for disembodied cultured networks to achieve functional plasticity. This neural plasticity provides a potential learning capability to cultured networks. Jimbo et al. [15] used a localized tetanic stimulus to induce long-lasting changes in the network responses that could be either potentiated or depressed depending on the electrode used to evoke the responses. Moreover, we and others previously found that such tetanus-induced plasticity was spatially localized and asymmetrically distributed [16],[17]. By delivering two different tetanic stimulation patterns, Ruaro et al. trained a cultured network to discriminate the spatial profiles of the stimuli. These results suggest that different stimulation patterns can shape diverse functional connectivity in cultured networks. By incorporating closed-loop feedback, Shahaf and Marom [18] showed unidirectional learning: to induce an electrode-specific increase in response. This simple form of learning was achieved by a binary training: to stop a periodic stimulation at one electrode when the desired response level at the target electrode was obtained. In order to scale to more complex behavior, we need to create more structured training stimuli and detailed activity metrics to investigate whether an embodied cultured network can learn multiple tasks simultaneously.

Unlike *in vivo* systems, the sensory-motor mapping and training algorithm in an embodied cultured network are defined by the experimenters. In order to efficiently find an effective closed-loop design among infinite potential mappings, we first embodied a biologically-inspired simulated network to study an adaptive goal-directed behavior in an animat: learning to move toward and stay within a user-defined area in a 2-D plane. The simulated network of 1000 leaky integrate-and-fire neurons expressed spontaneous and evoked activity patterns similar to that of the dissociated cortical cultures [19]. Furthermore, a similar but larger simulated network showed that localized coherent input resulted in shifts of receptive and projective fields similar to those observed *in vivo* [20]. Thus simulated networks show promise for analyzing biological adaptation with various closed-loop designs.

The closed-loop design we discuss here consists of four unique elements:

1. Patterned stimulation to induce network plasticity. This low-frequency (∼3 Hz) training stimulation differs from most studies of cultured networks, where plasticity was induced by high frequency tetanic stimulations [15],[17].
2. Continuous low-frequency background stimulation (∼3 Hz) to stabilize accumulated plasticity [19], which is analogous to continuous sensory inputs and ongoing processing in the brain.
3. Population coding for motor mapping. Population coding is considered a robust means to represent movement directions in the primary motor cortex [21].
4. Adaptive selection of training stimulation. Because the connectivity in a cultured network is not predictable, the effects of a given training stimulation cannot be known *a priori*. Thus we delivered training stimulation contingent on the animat's performance in order to direct changes in network connectivity that further shift the animat's behavior toward the desired behavior.

Here, we demonstrate adaptive goal-directed behavior in the simulated network, where multiple tasks were learned simultaneously. The desired behavior could only be achieved with proper selection of stimuli to encode sensory inputs and a variety of training stimuli with adaptive selection contingent on the animat's behavior.

While lacking the characteristic layered structure of *in vivo* cortical tissue, the biologically-inspired simulated network still could be functionally shaped, and showed meaningful behavior, demonstrating that these neural networks have an innate ability to process information. The proposed design is not restricted to a particular sensory-motor mapping, and could be applied with different and more complex goal-directed behaviors, which may provide a useful *in vitro* model for studying sensory-motor mappings, learning, and memory in the nervous system.

## Methods

We designed a closed-loop system consisting of an animat and a biologically inspired simulated network, looped together through the stimulation of virtual electrodes to encode sensory information and to direct learning, and through recordings from the virtual electrodes used to generate motor output. A series of experiments was performed to validate some of the designs, to determine the system's ability to learn a pre-determined goal behavior, and to verify what was essential in the system for successful learning.

### Closed-Loop System

#### Animat

##### Environment

**Figure 1:**
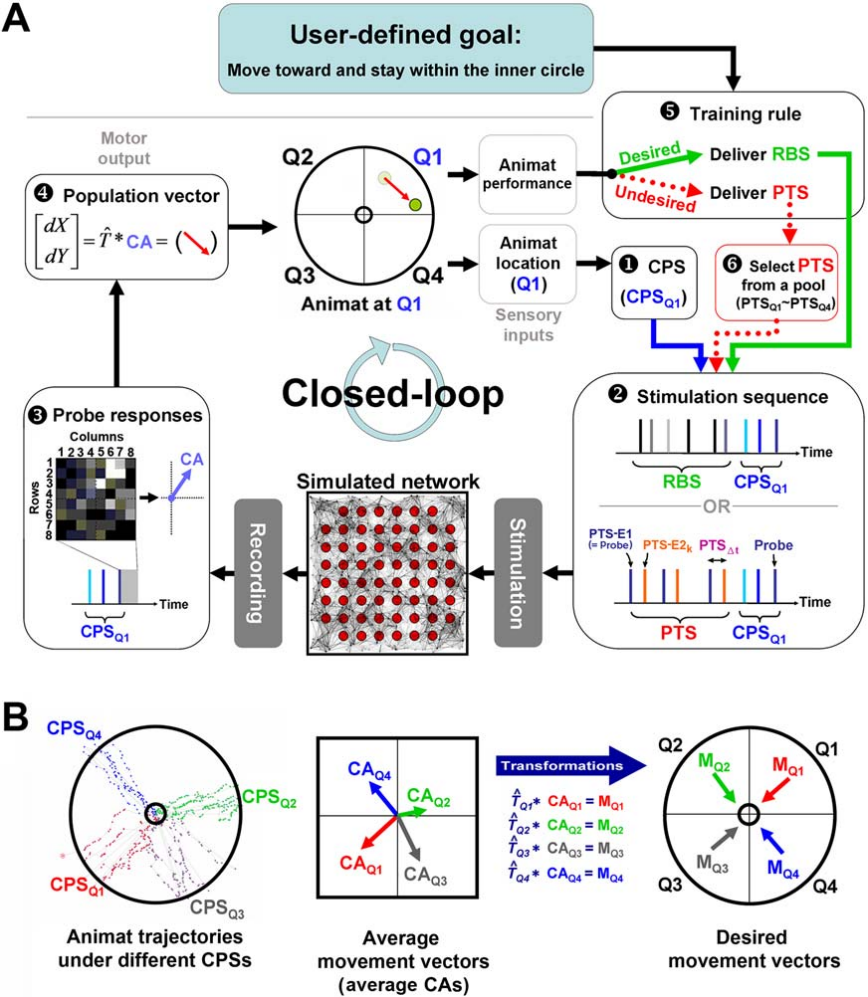
Closed-loop algorithm. (A) Closed-loop design: the sensory mapping (1–2), the motor mapping (3–4), and the training rules (5–6). Refer to Methods for a detailed explanation. (B) Motor mapping transformation. Left: In the beginning of each experiment, each CPS (CPS_Q1_–CPS_Q4_) was continuously delivered every 5 seconds with RBS in between. After the animat reached the outer circle, it was moved back to the inner circle. Middle: The average CAs from probe responses to each CPS were calculated (CA_Q1_–CA_Q4_). The average CAs represent the average movements from each CPS. Right: The transformation 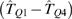 for each CPS was created so that the average movement in each quadrant would be the desired movement with a magnitude of 1 unit (M_Q1_–M_Q4_).

The animat was controlled by a simulated network (see Biologically inspired simulated network section below) to move in a plane within a circle of 50 units radius, which was divided into four quadrants (Q1: northeast, Q2: northwest, Q3: southwest, and Q4: southeast, see Figure 1A). The animat was put back to a random location within a smaller concentric circle of 5 units radius if it moved outside the outer circle.

##### Goal

The goal of the animat was to move and stay within a smaller concentric circle of 5 units radius (see Figure 1A). Successful behavior required that animat movement in each quadrant be towards the origin.

##### Sensory system and motor capability

The animat had two sensory inputs and the neural network's response to the first determined animat movement (Figure 1A).

###### Animat location

Location was one of four discrete values representing which quadrant the animat was in (Q1–Q4). Sensory input was applied to the neural network every 5 seconds by stimulating a corresponding sequence of electrodes (CPS_Q1_–CPS_Q4_; see Stimulation protocols section below). The last electrode in the sequence was termed “probe” and evoked network responses used to determine animat movement (see Motor mapping section below).

###### Animat performance

If the animat was outside of the inner circle, its performance determined whether training was required (see Training rules section below). Patterned training stimuli (PTS; see Stimulation protocols section below) was applied if the animat was moving away from the inner circle in order to cause neural plasticity and induce learning. Otherwise, the goal-behavior was being achieved, and random background stimulation (RBS; see Stimulation protocols section below) was applied in order to maintain animat behavior. In order to acquire sufficient training between two movements, the sensory input of location (and thus animat movement) was evaluated every 5 seconds.

#### Biologically inspired simulated network

The animat was connected to a simulated network through a sensory-motor loop (Figure 1A). We used the Neural Circuit SIMulator [22] to produce three artificial neural networks, described previously [19] with parameters detailed in Supplemental Material Text S1. Briefly, 1,000 leaky integrate-and-fire (LIF) model neurons, with a total of 50,000 synapses, were placed randomly in a 3 mm by 3 mm area. All synapses were frequency-dependent [20],[23] to model synaptic depression. Seventy percent of the neurons were excitatory, with spike-timing-dependent plasticity (STDP) [24]. We included an 8 by 8 grid of electrodes, 60 of these (see Figure 1A, red circles in the simulated network) were used for recording and stimulation as in a typical real multi-electrode array (MEA) used in our lab (from Multi Channel Systems). The networks were run without external stimulation for 5 hours in simulated time and then with random background stimulation (RBS, see below) for another two hours until the synaptic weights reached equilibrium. The set of stabilized synaptic weights was used as the initial state for the corresponding network.

In a previous study, we showed that our 1000-neuron LIF model and living MEA cultures expressed similar spontaneous and evoked activity patterns, demonstrating the usefulness of the LIF model for representing the activity of biological networks [19]. In another study, we successfully used this simulated network to find a statistic to detect network functional plasticity in living MEA cultures and to demonstrate region-specific properties of stimulus-induced network plasticity [16].

#### Closed-loop algorithm

The closed-loop design in this work included (1) three different stimulation protocols encoding sensory inputs, inducing learning, and maintaining what was learned, (2) a simple sensory mapping, (3) a motor mapping with population coding incorporating spatial information of network activity, and (4) training rules with adaptive selections of training stimuli.

##### Stimulation protocols

We used three classes of stimulation protocols for three different purposes: (1) Four *context-control probing sequences (CPSs)* (CPS_Q1_–CPS_Q4_) were used to encode 4 sensory inputs (current location = Q1-Q4). These also evoked neural activity used as motor commands for the animat. (2) Four “pools” of *patterned training stimulation (PTS)* (PTS_Q1_–PTS_Q4_), each also assigned to Q1-Q4, were used to induce network plasticity to train the animat. (3) *Random background stimulation (RBS)* was used to stabilize accumulated plasticity, and was shown previously to stabilize network synaptic weights [19].

###### Context-control probing sequence (CPS)

Four stimulation sequences were used (CPS_Q1_–CPS_Q4_). Each CPS consisted of a sequence of 3 stimulation pulses from 3 randomly selected electrodes with inter-pulse intervals randomly selected between 200 to 400 msec (Figure 1A). The last stimulus, termed probe, was unique to each CPS. For each experiment, the CPSs were fixed throughout.

Each CPS (CPS_Q1_- CPS_Q4_) was delivered every 5 seconds, when the corresponding sensory input (Q1- Q4) was evaluated. We used the evoked action potentials from the last stimulus (probe responses) to generate motor commands to control the animat. The context before the probe stimulus was found to influence the probe response [25]. Therefore, in order to directly quantify learning by changes in movement, we sought to reduce the variability in the probe response due to recent neural activity and stimulation history, such that changes in probe responses were due mainly to changes in network connectivity. We found that by controlling the stimulation context (the first two stimuli of a CPS) before the probe with inter-pulse intervals between 200 to 400 msec, the variability of the probe responses was minimized. Data supporting this in both simulated and living networks are shown in Supplemental Material Text S2.

###### Patterned training stimulation (PTS)

Four pools of PTSs (PTS_Q1_–PTS_Q4_) were used, each associated with its corresponding quadrant. A PTS consisted of repetitive stimulation at two electrodes. The location of the first electrode (PTS-E1) was chosen as the probe electrode used in the preceding CPS (for PTS_Q1_, it was the last stimulus in CPS_Q1_). The two parameters varied among different PTSs in a pool were: the location of second electrode (PTS-E2_k_), and the relative timing from the first electrode (inter-pulse interval, PTS_t_) (see Figure 1A). PTS-E2_k_ was chosen from one of the 60 electrodes (k = 1–60), and PTS_t_ was chosen from one of 11 values: −100, −80, −60, −40, −20, 0, 20, 40, 60, 80, and 100 msec. Therefore, each pool consisted of 660 ( = 60*11) PTSs.

During training, a PTS was delivered repetitively at the pair of electrodes with random inter-PTS-intervals between 400 to 800 msec. Paired stimulation of monosynaptically connected neurons evokes STDP dependent on the stimulation interval [26], and paired stimulation of two electrodes has the potential to induce STDP throughout any shared activation pathways in the network. In our simulated networks, we found that the network could be shaped into a variety of possible synaptic states by using paired stimulation with different stimulation parameters (electrode pairs, inter-PTS-intervals, etc.) (data not shown). This validates the use of PTSs to direct network plasticity.

###### Random background stimulation (RBS)

RBS was delivered randomly at 60 electrodes, one at a time, with random inter-pulse-intervals ranging from 200 to 400 msec (see Figure 1A). RBS of an aggregated frequency of 1 Hz was shown previously to have stabilizing effects on network synaptic weights in a simulated network after stimulus-induced plasticity [19]. Thus we delivered RBS to maintain the network synaptic weights if the desired behavior was observed. In this study, the aggregated stimulation frequency of RBS was increased to 3 Hz so that amounts of stimulation in RBS and PTS were comparable.

The closed-loop system consisted of three parts (see Figure 1A): the sensory mapping, the motor mapping, and the training rules.

##### Sensory mapping

One CPS (CPS_Q1_, CPS_Q2_, CPS_Q3_, or CPS_Q4_) was delivered every 5 seconds based on which sensory input was received (Q1, Q2, Q3, or Q4) (1 and 2 in Figure 1A).

##### Motor mapping: Center of activity (CA)

After delivering a CPS, the number of spikes within 100 msec after the probe were measured at 60 recording electrodes, and the Center of Activity (CA) was calculated (3 in Figure 1A) [19]. CA represents the spatial asymmetry of the activity, which is analogous to the center of mass. Assume FR(k) represents firing rates at recording electrode k within 100 msec after the probe, and Col(k) and Row(k) are the column number and the row number of electrode k, which range from 1 to 8. For example, electrode 28 has column number 2 and row number 8 (see 3 in Figure 1A). Then CA is a two dimensional vector:
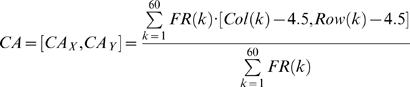
(1)where [4.5, 4.5] represents the center of the 8 by 8 grid of electrodes. Previously we found that the network synaptic state could be more effectively decoded by incorporating the spatial information of activity distribution [16].

##### Motor mapping: Population coding and motor mapping transformation

We instructed incremental movement of the animat [*dX*, *dY*] by using a population vector calculated from CA (4 in Figure 1A):
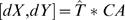
(2)where 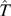 is a transformation matrix that transformed CAs in the four quadrants into desired movements with average 1 unit moving distance.

In the beginning of each experiment, CPS_Q1_ was continuously delivered every 5 seconds with RBS in between. After the animat reached the outer circle, it was moved back to the inner circle, and CPS_Q2_ was delivered, then CPS_Q3_ and CPS_Q4_. The whole process was repeated 5 times, and the average CAs from probe responses to each CPS were calculated (shown as CA_Q1_–CA_Q4_ in Figure 1B). The average CAs represent the average movements from each CPS. The transformations 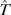 for each CPS were created so that the average movement in each quadrant would be the desired movement (M_Q1_–M_Q4_; pointing to the center of the inner circle) with a magnitude of 1 unit (see Figure 1B). For example, for CA_Q1_ = [*CA_Q1,X_, CA_Q1,Y_*] and the desired movement 
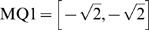
, the transformation 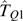 consisted of two scaling numbers *α_Q1_, and β_Q1_* that satisfied:
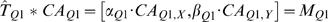
(3)Thus, for a CPS_Q1_ delivered with no neural plasticity, the animat will move on average at a −135° angle by 1 unit distance. For each experiment, the transformations 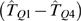 were calculated first, and then fixed for the duration of the experiment.

##### Training rules

If the animat's performance was desirable (moving inward), then RBS was delivered for 5 seconds until the next sensory input was evaluated (5 to 2 in Figure 1A). If the animat's performance was not desired (moving outward), then training was applied (5 in Figure 1A): a PTS was randomly selected from the corresponding pool; if the previous CPS was CPS_Q1_, then the PTS was selected from PTS_Q1_ (6 in Figure 1A) and delivered for 5 seconds (2 in Figure 1A). If the performance of the animat was improved but still not desirable after the PTS (still moving outward but at a slower rate), then the same PTS would be used for the next training. Initially, the probability of choosing a PTS from a pool was identical (1/660). Every time a PTS improved the performance of the animat after the next probe, a copy was added into its pool. Thus the size of the pool increased, and the probability of this “favorable” PTS being chosen later was increased. In contrast, if that PTS worsened the performance of the animat (moving outward faster), it was removed from the pool, unless only one PTS of this specific type remained.

To summarize, if the animat was moving correctly, RBS was delivered to stabilize the corresponding network synaptic state. Otherwise, PTS was delivered to change the network synaptic weights. Also, the probability of specific PTS patterns being chosen was constantly updated according to the performance of the animat.

### Simulation Experiments

We used three networks with different connectivity, each with 5 different sets of CPSs (randomly selected CPS_Q1_–CPS_Q4_). These 15 setups with different network connectivity and sensory-motor mappings were used for the following simulation experiments:

#### Experiment 1: Validate effects of RBS on stability of network input-output functions

This experiment was performed to validate the design of using RBS to maintain the desired behavior. In a previous study, we showed that RBS helped stabilize network synaptic weights after stimulus-induced plasticity in a simulated network [19]. Here we further verified how this effect on network synaptic weights affected stability of the network input-output function, that is, stability of the animat's movement under the same sensory input.

The animat was run with RBS between CPSs without training (no PTS) for one hour. We compared this to the animat's performance without RBS (CPSs only). The initial network state, the random seed for fluctuations in neurons' membrane potentials and synaptic currents, and the sensory-motor mapping were not varied.

We used mutual information to quantify stability of the relation between sensory inputs (discrete values of 1, 2, 3, or 4 for Q1, Q2, Q3, or Q4, respectively) and motor outputs (animat's movement angles from −180° to 180). Mutual information is a better quantity to measure the general dependence between stimuli (sensory inputs) and responses (motor outputs) than the correlation function which only measures the linear dependence [27]. Furthermore, mutual information can be applied to symbolic sequences, such as discrete values of sensory inputs here, while the correlation function can be only applied to numerical sequences [27]. The animat's sensory inputs (Q1, Q2, Q3, or Q4) and movement angles (−180–180) were recorded and mutual information was calculated in 5-min moving time windows with a time step of 5 seconds using the histogram-based mutual information methods [28]. The higher the mutual information between sensory inputs and motor outputs, the lower the uncertainty about the sensory input after a motor output is observed, that is, the higher the stability of the animat's movement under the same sensory input.

#### Experiment 2: Quantify learning by switching the sensory mapping

We investigated the networks' ability to learn a user-defined goal behavior by “switching” the sensory mapping. This would be analogous to placing an animal into a different environment, or imposing a new task. As described previously, the sensory-motor mapping was set up so that the animat would move toward the center as desired. We quantified the animat's ability to adapt to a switch of the sensory mapping, that is, the ability to restore desired behavior under a different sensory mapping.

The transformation, 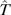, allowed the animat to move correctly, on average, and after 10 minutes the sensory mapping was switched by exchanging CPS_Q1_ and CPS_Q3_ while CPS_Q2_ and CPS_Q4_ remained unchanged. That is, if the animat was at Q1, CPS_Q3_ was delivered instead of CPS_Q1_, and vice versa. The simulation was stopped when either the simulation time exceeded 4 hours without reaching the goal or the animat stayed within the inner circle 90% of the time (reached the goal) for 10 minutes. If the animat was able to adapt to the new sensory mapping and learn the desired behavior, the network was considered successfully rewired. The time course of this adaptation was quantified by the learning curve, which was measured as the probability of successful behavior within a 2-min moving time window with 5-sec step.

#### Experiment 3: Avoid unsuccessful learning by selecting CPSs with small *Max(CA_Q1_, CA_Q3_)* and small *Max overlap*

In order to avoid unsuccessful adaptations, we selected CPSs that evoked less localized and less overlapped responses (see Results), instead of random selections used in Experiment 2. The level of localization in responses was quantified by *Max(CA_Q1_, CA_Q3_)*, which was the maximum of CA_Q1_ and CA_Q3_ (average CAs to CPS_Q1_ and CPS_Q3_). The reason that only responses to CPS_Q1_ and CPS_Q3_ were used are described in Results. The degrees of overlap between the responses of different pairs of CPSs were quantified by *Max overlap*. Assume that *N_Q1_* is the set of neurons activated by CPS_Q1_, and *N_Q2_* is the set of neurons activated by CPS_Q2_. Then the degree of overlap between responses to CPS_Q1_ and CPS_Q2_ was defined as:
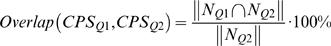
(4)where ||·|| represents the number of elements in the set. This value indicates the proportion of neurons activated by CPS_Q1 _that were also activated by CPS_Q2_, which quantifies how much the training in Q1 (a switched quadrant) might affect the behavior in Q2 (un-switched). The maximum of all possible overlaps between a switched quadrant and an un-switched quadrant was found:
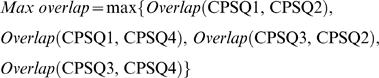
(5)We randomly generated 85 sets of CPSs, in addition to the 15 original ones, and randomly selected 10 sets that satisfied the criteria of *Max(CA_Q1_, CA_Q3_)<150* and *Max overlap<50%*. Then we repeated Experiment 2 with these 10 setups to see whether the success rate of adaptations could be improved.

#### Experiment 4: Verify the contribution of the network to learning in the system

The selection of PTSs was an adaptive process. Therefore, successful adaptations in the behavior of the system could solely be a product of the artificial adaptive training algorithm. In order to verify whether the network had contributed toward learning, we repeated the successful-learning simulations found in Experiment 2 with the STDP algorithm turned off to see whether successful adaptations remained. In each new simulation, the same random seed, the same initial network synaptic weights, the same sensory-motor mappings, and the same simulation duration were used as in the corresponding original one. This was analogous to applying neurotransmitter receptor antagonists, such as APV, to block synaptic plasticity in the culture. If learning degrades without the STDP algorithm, then network plasticity is contributing to successful adaptation.

#### Experiment 5: Verify the importance of availability of different PTSs

We hypothesized that the same PTS might have different effects at different points in time, and therefore successful adaptations would require a variety of different PTSs (see Results). In order to verify this hypothesis, we repeated the successful-learning simulations found in Experiment 2, but used only one PTS pattern for training in each quadrant instead of a pool of 660 PTSs as before. In order to increase the likelihood that these PTSs could achieve better learning results, we selected the four most frequently used PTSs, one for each quadrant in the original successful-learning simulation. A new simulation was run with the same random seed, the same initial network synaptic weights, the same sensory-motor mappings, and the same simulation duration, as in the original simulation.

#### Experiment 6: Verify the importance of behavior-contingent training

In order to verify the importance of behavior-based training on the performance of the animat, we recorded the whole training stimulation sequence (PTS and RBS) for each successfully adapted simulation in Experiment 2 and replayed it into the same network with the same initial state and with the same sensory-motor mapping. In the replayed-training simulation, a different random seed for fluctuations in neurons' membrane potentials and synaptic currents was used. Thus, responses to CPSs in the replayed-training simulation were not identical to those in the original successful-learning simulation, and hence the trajectory of the animat rapidly diverged from that of the original simulation. The replayed training stimulation was delivered regardless of whether the movement was desired or not. Therefore, the training stimulation soon became no longer contingent on the network activity.

#### Experiment 7: Verify the uniqueness of “solutions”

In order to investigate whether under a specific sensory mapping, the desired behavior could only be exhibited by a specific set of network synaptic weights, we switched the sensory mapping back to the original sensory mapping, after the network adapted to the switched sensory mapping in Experiment 2, to see whether the network could re-adapt to the original mapping. If the network was able to re-adapt to the original mapping, we checked whether the network synaptic weights were the same as the first time.

## Results

In order to investigate how external training stimuli can shape a network into a desired state, we used a biologically-inspired simulated network to study multi-task goal-directed behavior by embodying the network with an animat. We first validated the design of using random background stimulation (RBS) to maintain what was learned (Experiment 1). We then quantified the system's learning ability (Experiment 2), and investigated the reasons for unsuccessful learning (Experiment 3). We showed that learning in the network was responsible for successful learning in the overall closed-loop system (Experiment 4), and further verified the importance of using a sequence of PTS patterns for training (Experiment 5) contingent on behavior (Experiment 6). We finish by demonstrating that the same desired behavior could be exhibited with different sets of network synaptic strengths (Experiment 7). Experiment protocols are further detailed in Methods. All acronyms are shown in Table 1. A diagram of the closed-loop system, stimulation sequences, and motor transformations is shown in Figure 1.

**Table 1:** Acronym list.

Abbreviation		Full Name
Stimulation protocol	CPS	Context-control probing sequence
	PTS	Patterned training stimulation
	RBS	Random background stimulation
PTS parameters	PTS_Δt_	Inter-pulse interval of a PTS
	PTS-E1	First electrode in a PTS ( = probe electrode)
	PTS-E2_k_	Second electrode in a PTS
CA		Center of activity
MEA		Multi-electrode array
STDP		Spike-timing-dependent plasticity

### Experiment 1: Random Background Stimulation (RBS) Helped Maintain the Network Input-Output Function

**Figure 2:**
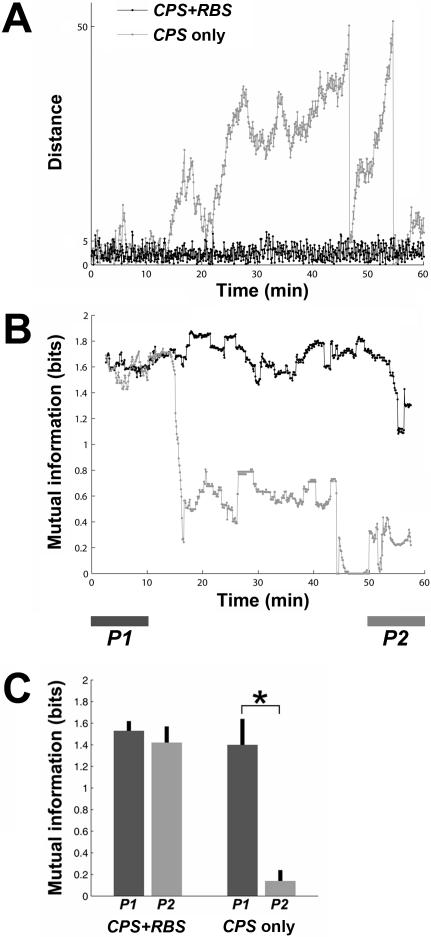
RBS stabilized the network input-output function. (A) An example of the time course of the distance between the animat and the origin. The animat stayed within the desired area (the inner circle of 5 units radius) for more than 95% of an hour when RBS was applied. When no RBS was applied, the animat moved outward after 10 minutes. When the animat reached the outer circle of 50 units radius, it was put back to a random location within the inner circle, which is shown as vertical downward lines. (B) The mutual information between the movement angle and the sensory input. When no RBS was applied, the mutual information decreased significantly when the animat started moving outward. (C) Comparison between the mutual information during the last 10 minutes (light gray, *P2* period shown in [B]) and that during the first 10 minutes (dark gray, *P1*) for the 15 simulations (3 networks, 5 different selections of CPSs each). With RBS, the mutual information in *P2* was comparable to that in *P1* (p = 0.77). Without RBS, the mutual information in *P2* was significantly lower than that in *P1* (p<1e-4, shown as an asterisk).

In order to validate the use of RBS to maintain desired behavior, the animat was run with RBS between context-control probing sequences (CPSs) without training (no PTS), and the results were compared to the animat's performance without RBS (CPSs only). An example of the time course of the animat's distance from the origin is shown in Figure 2A. The motor mapping was transformed (by 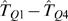, see Figure 1B) to obtain desired movements before the simulation. Therefore, in the beginning of both simulations with RBS and without RBS, the animat moved in desired directions in each quadrant and stayed within the inner circle. The animat maintained this desired behavior for the entire hour over 90% of the time when RBS was applied, whereas it moved outward after 10 minutes when no RBS was applied.

The mutual information between the movement angle and the sensory input is shown in Figure 2B. When the animat started moving outward in an undesired direction, the mutual information decreased significantly. This indicates decreasing stability of the animat's movement under the same sensory input. The mutual information during the last 10 minutes (*P2* period in Figure 2B) was compared to the mutual information during the first 10 minutes (*P1*) in the 15 simulations (3 networks, 5 different selections of CPSs each) (Figure 2C). With RBS, the mutual information in *P2* was 1.42±0.15 bits (mean±SEM, n = 1800 measures, 15 networks, 120 measures in 10 min per network), which was comparable to 1.53±0.09 bits in *P1* (p = 0.77, Wilcoxon signed-rank test). Without RBS, the mutual information in *P2* was 0.14±0.10 bits, which was significantly lower than 1.40±0.24 bits in *P1* (p<1e-4). This indicates that RBS with an aggregate frequency of 3 Hz maintained stability of the network input-output function, validating the use of RBS to maintain desired behavior in the animat. Furthermore, the results also suggested that repetitive non-training stimuli (CPSs and RBS) were unable to induce enough plasticity to systematically alter the animat's behavior.

### Experiment 2: Adaptation to the Switched Sensory Mapping

**Figure 3:**
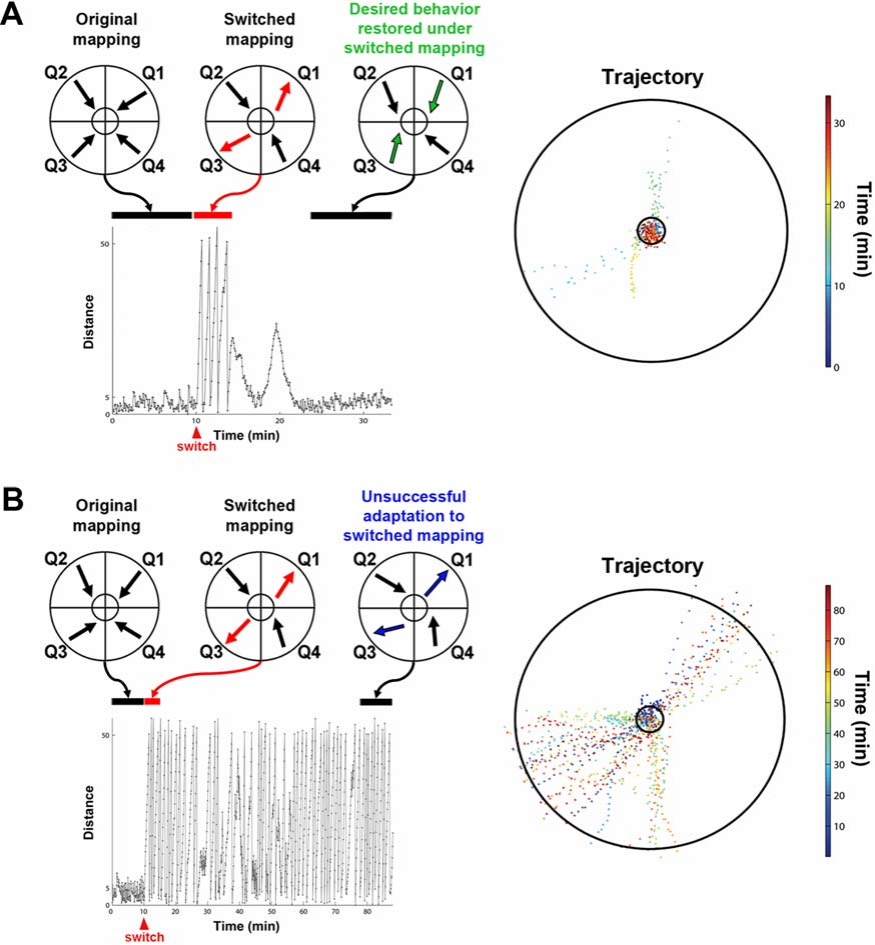
Adaptation to a new sensory mapping. The animat's learning ability was quantified by its ability to restore desired behavior after a sensory mapping switch. (A) An example of successful learning. The distance between the animat and the origin is shown in the left panel. The animat maintained the desired behavior for the first 10 minutes (the average inward movement in each quadrant during this 10-min duration is shown on the top), before the sensory mapping switch was performed between quadrants Q1 and Q3 at 10 minutes into the simulation. Immediately after the switch, the animat started moving outward (the trajectory is shown in the right panel). The red arrows on the top indicate the average outward movements in Q1 and Q3 during a 5-min time bin after the switch. Eventually, the animat adapted to the switch and restored the desired behavior to stay within the inner circle under the new sensory mapping. The average movements in all quadrants became toward the center again during the last 10 minutes, where the restored desired movements in Q1 and Q3 are highlighted in green. Ten simulations (out of 15) showed successful adaptation to the switch. (B) An example of unsuccessful learning. The animat kept moving outward and was repeatedly returned to the inner circle after reaching the outer circle. The training was unable to restore the desired behavior throughout 4 hours of experiment. Only the first 90 minutes are shown for clarity. One-third of the simulations showed unsuccessful learning.

**Figure 4:**
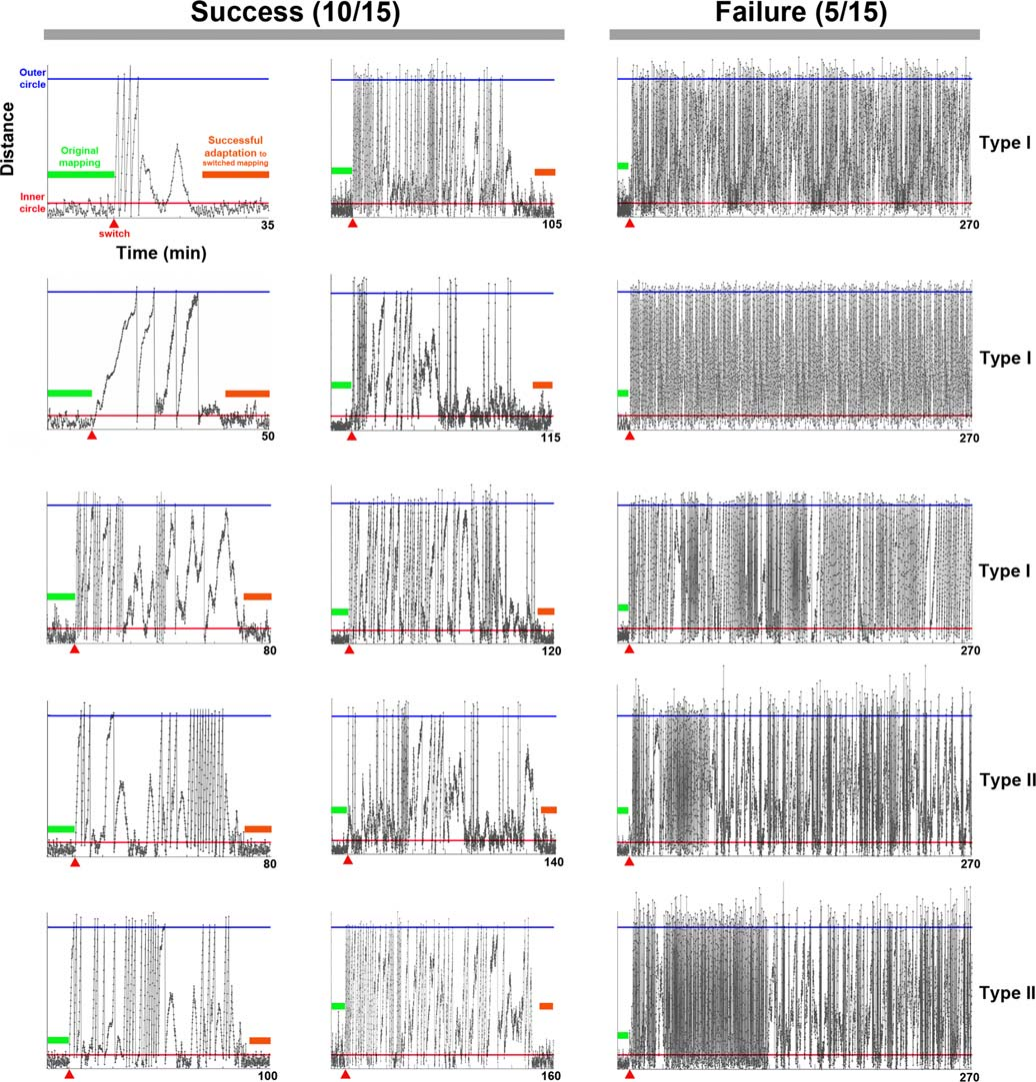
All successful and unsuccessful learning simulations. The distances between the animat and the origin in all 15 simulations are shown. The animat maintained the desired behavior before the sensory mapping switch (red triangle) between quadrants Q1 and Q3 at 10 minutes into the simulation (green bar). Immediately after the switch, the animat started moving outward. In 10 simulations, the animat adapted to the switch and restored the desired behavior to stay within the inner circle under the new sensory mapping (orange bar). For the other 5 with unsuccessful learning, the animat kept moving outward and was repeatedly returned to the inner circle after reaching the outer circle. The training was unable to restore the desired behavior throughout 4 hours of experiment (only the first 3 hours are shown for clarity). Type I and Type II failures are indicated (see Results).

We investigated the networks' ability to learn a user-defined goal behavior by “switching” the sensory mapping. A motor mapping was created (through transformations 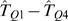) to obtain desired movements before the experiment began (Figure 1B). The animat's performance was observed for 10 minutes, demonstrating robust goal-directed behavior (Figures 3 and 4). Then the sensory mapping was suddenly and drastically altered, so that the animat's behavior was no longer correct. Specifically, a CPS appropriate for evoking movement toward the center from Q1 was now delivered when the animat was in Q3, and vice versa. Learning was then quantified by the animat's ability to adapt to the new, fixed sensory mapping and exhibit goal-seeking behavior.

Ten simulations, out of 15, showed successful adaptation to the switch. One successful simulation is shown in Figure 3A, and the corresponding movie is shown in Supplemental Material Movie S1. Immediately after the switch, as expected, the animat moved outward in the quadrants where the sensory mapping switch was performed (Q1 and Q3). Patterned training stimulation (PTS), paired stimulation designed to induce STDP throughout any shared activation pathways in the network, began to shape the network synaptic weights, and the desired behavior was restored under the switched mapping. An unsuccessful simulation is shown in Figure 3B. In 5 unsuccessful simulations, the animat kept moving outward and was repeatedly put back into the inner circle whenever it reached the outer circle. The training was unable to restore the desired behavior throughout a 4-hr simulation. In Figure 3B, only the first 90 minutes are shown for clarity.

Distance plots for all 15 simulations are shown in Figure 4. For successful simulations, the average time for the adaptation was 88.6±12.2 minutes (mean±SEM, n = 10 successful-learning simulations). Two different types of unsuccessful learning are also indicated (Type I and Type II failures, see below).

### Experiment 3: Avoid Unsuccessful Learning by Selecting Stimuli to Encode Sensory Inputs

One-third of the simulations showed unsuccessful learning but were nevertheless informative (see Figure 4). Two types of failures were observed in these following 5 unsuccessful experiments.

#### Type I failure

**Figure 5:**
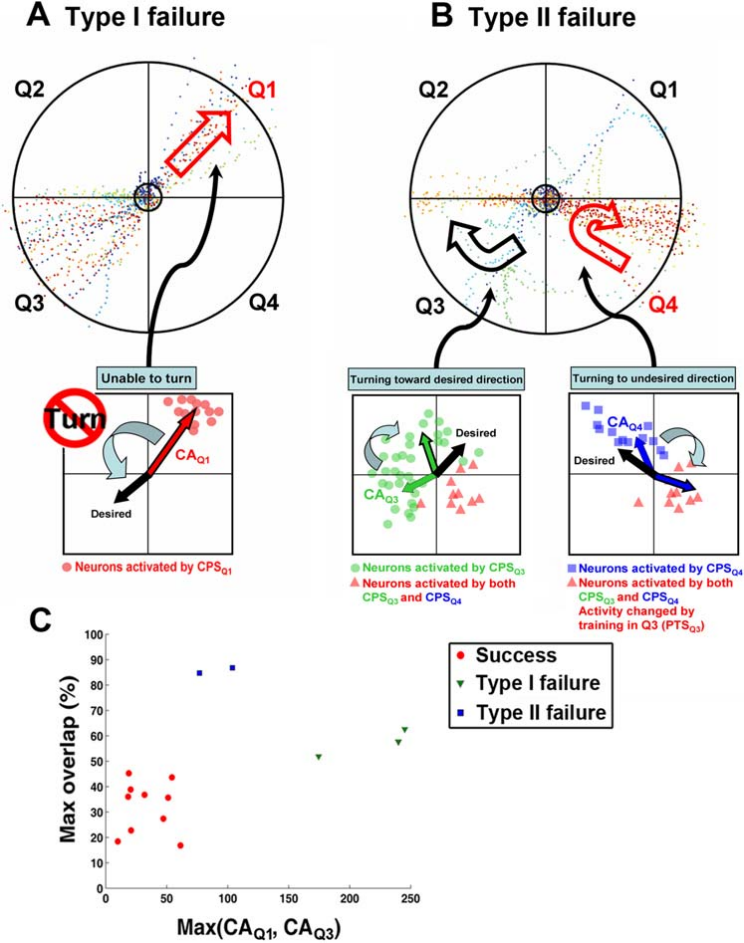
Hypotheses about the reasons for unsuccessful learning. One-third of the experiments showed unsuccessful learning. Two types of learning failures were found, and examples are shown. (A) Type I failure: the animat showed no sign of improving behavior in the quadrant(s) where the switch of the sensory mapping was performed (Q1 and/or Q3). Using the trajectory in Q1 as an example, the animat kept going outward without turning (indicated as a hollow red arrow). In those cases, CPS_Q1_ and/or CPS_Q3_ evoked activity in neurons localized mainly at one quadrant of the network. The localization of neurons activated by CPS_Q1_ is illustrated in the cartoon. We hypothesize that this localization reduced or eliminated the ability of the responses to shift the CA from the original direction (shown as a solid red arrow) toward the desired direction (shown as a black arrow). (B) Type II failure: the animat showed signs of improving by changing movement direction(s) in the quadrant(s) where the switch was performed (Q1 and/or Q3). However, the original desired movement direction(s) in the un-switched quadrant(s) (Q2 and/or Q4) was/were changed into undesired ones(s). Using the trajectory in Q3 and Q4 as an example, the animat was able to turn in Q3 (shown as a hollow black arrow) but the desired direction in Q4 was later altered (shown as a hollow red arrow). In those cases, neurons activated by different CPSs had large degrees of overlap. The neurons activated both by CPS_Q3_, CPS_Q4_, and both are illustrated in the cartoon. We hypothesize that the training stimuli in Q3 caused correlated changes in the overlapped neurons (shown as red dots), which caused undesired change in responses to CPS_Q4_. (C) The degree of overlap (quantified by *Max overlap*, see Methods) is plotted versus the degree of localization (quantified by *Max(CA_Q1_, CA_Q3_)*), which shows that smaller overlap, smaller CA_Q1_ and smaller CA_Q3_ were found in all 10 successful cases. Also, Type I failure showed large *Max(CA_Q1_, CA_Q3_)* and Type II failure showed large *Max overlap*.

The animat showed no sign of improving behavior in the quadrant(s) where the switch of the sensory mapping was performed (Q1 and/or Q3) (see Trajectory in Figure 5A). In those cases, CPS_Q1_ and/or CPS_Q3_ evoked activity in neurons localized mainly at one quadrant of the network. We hypothesized that this localization reduced or eliminated the ability of the responses to shift the direction of the CA, and thus movement could not be shifted toward a different direction. Compared to more spatially homogeneous or symmetric responses, a localized response results in a larger magnitude in CA (see Equation 1 in Methods). Therefore, we used *Max(CA_Q1_, CA_Q3_)* to quantify the level of localization in responses to CPS_Q1_ and CPS_Q3_ (see Methods). This measure indicates the likelihood that the directions of CAs to CPS_Q1_ and CPS_Q3_ can be “reversed”.

#### Type II failure

The animat showed signs of improving by changing moving direction(s) in the quadrant(s) where the switch was performed (Q1 and/or Q3). However, the movement direction in an un-switched quadrant (Q2 and/or Q4) became undesired (Figure 5B). In those cases, neurons activated by different CPSs had large degrees of overlap. We hypothesized that the training stimuli caused correlated changes in multiple CPSs. We used *Max overlap* to quantify the degrees of overlap between the responses of different pairs of CPSs (see Methods).

*Max overlap* is plotted versus *Max(CA_Q1_, CA_Q3_)* in Figure 5C, which shows that smaller overlap, smaller CA_Q1_ and smaller CA_Q3_ were found in all 10 successful-learning experiments. Also, as hypothesized, Type I failure showed large *Max(CA_Q1_, CA_Q3_)* and Type II failure showed large *Max overlap*.

**Figure 6:**
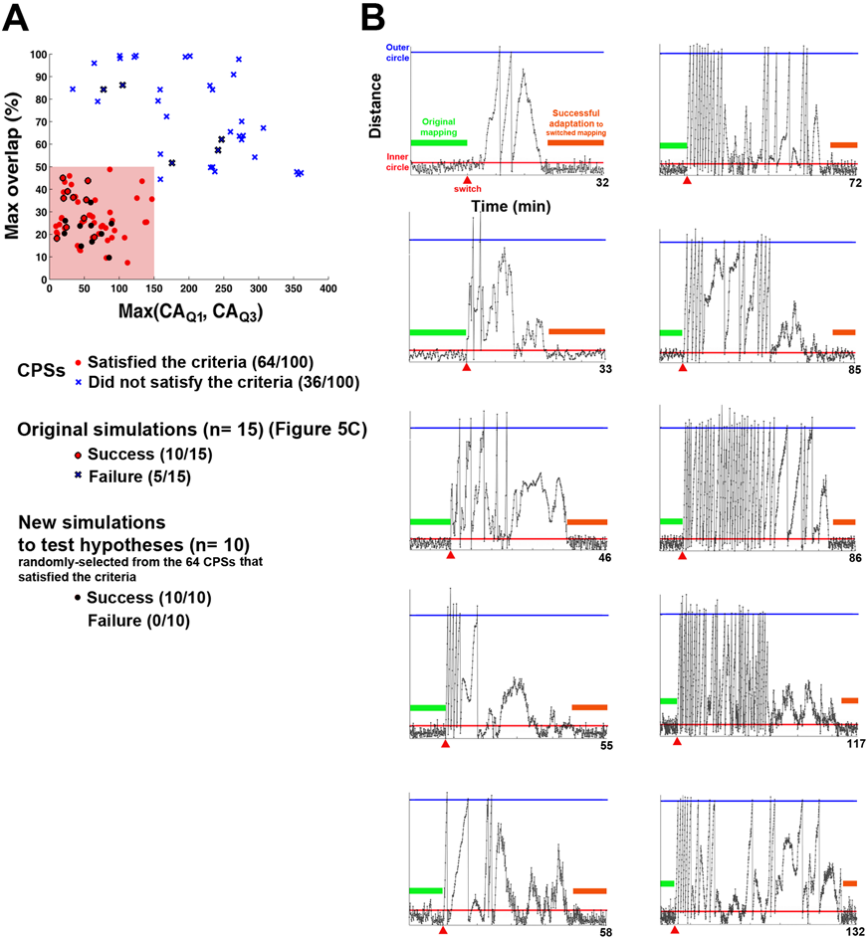
Improved learning by selecting CPSs based on the hypotheses. Successful adaptations can be achieved by selecting CPSs with small *Max(CA_Q1_, CA_Q3_)* and small *Max overlap*. (A) *Max(CA_Q1_, CA_Q3_)* and *Max overlap* from 100 randomly-selected sets of CPSs in the three simulated networks. The 15 sets of CPSs used in the previous simulations are indicated as dots and crosses with black outlines. Among the 100 sets, 64 sets satisfied the criteria of *Max(CA_Q1_, CA_Q3_)<150* and *Max overlap<50%* (red dots). (B) Successful learning was achieved by using 10 randomly-selected sets of CPSs that satisfied the criteria (the selections are indicated as black dots in [A]). The success rate was improved from 66.7% (10/15, see Figure 4) to 100% (10/10). The same representations are used as in Figure 4.

In order to further verify the hypotheses, we randomly generated additional 85 sets of CPSs for the 3 networks (a total of 100 sets including the 15 sets in the original simulation in Experiment 2), and randomly chose 10 sets with small overlap, small CA_Q1_ and small CA_Q3_ to repeat Experiment 2. The *Max(CA_Q1_, CA_Q3_)* and *Max overlap* of these 85 sets and the 15 sets used previously are shown in Figure 6A. A cluster with small *Max(CA_Q1_, CA_Q3_)* (<150) and small *Max overlap* (<50%) was observed (the shaded area in Figure 6A). Therefore, we hypothesized that Type I and Type II learning failures could be avoided by selecting CPSs within this cluster:

1. Type I failure can be prevented by choosing CPS_Q1_ and CPS_Q3_ that each evoke responses which are not too localized, (criterion: *Max(CA_Q1_, CA_Q3_)<150*).
2. Type II failure can be prevented by choosing CPSs that evoke responses without too much overlap, (criterion: *Max overlap<50%*).

Sixty-four out of the 100 sets of CPSs satisfied the criteria of *Max(CA_Q1_, CA_Q3_)<150* and *Max overlap<50%*. By using 10 randomly-selected sets of CPSs that satisfied the criteria to run 10 additional simulations, we found that successful learning could be reliably achieved (Figure 6B). The success rate was improved from 66.7% (from the 15 original simulations, see Figure 4) to 100% (from the 10 new simulations, Figure 6B). The chance that randomly selecting 10 CPSs that all satisfy the criteria from the 100 randomly generated sets is less than 0.01 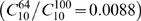. This supports the hypotheses and indicates that a higher success rate of adaptations can be achieved by selecting CPSs with smaller *Max(CA_Q1_, CA_Q3_)* and smaller *Max overlap*. The average time for the adaptation in these additional simulations was 71.8±10.7 minutes (n =  10 successful-learning simulations), which was comparable to 88.6±12.2 minutes in the 10 successful-learning simulations shown previously (p = 0.43, Wilcoxon rank sum test). Furthermore, 64 out of 100 random selections of CPSs (64%) satisfied the criteria (see Figure 6A), which was comparable to the success rate (66.7%) from the previous 15 simulations with CPSs selected randomly without the criteria.

### Experiment 4: Network Plasticity Was Essential for Successful Adaptations in the System

**Figure 7:**
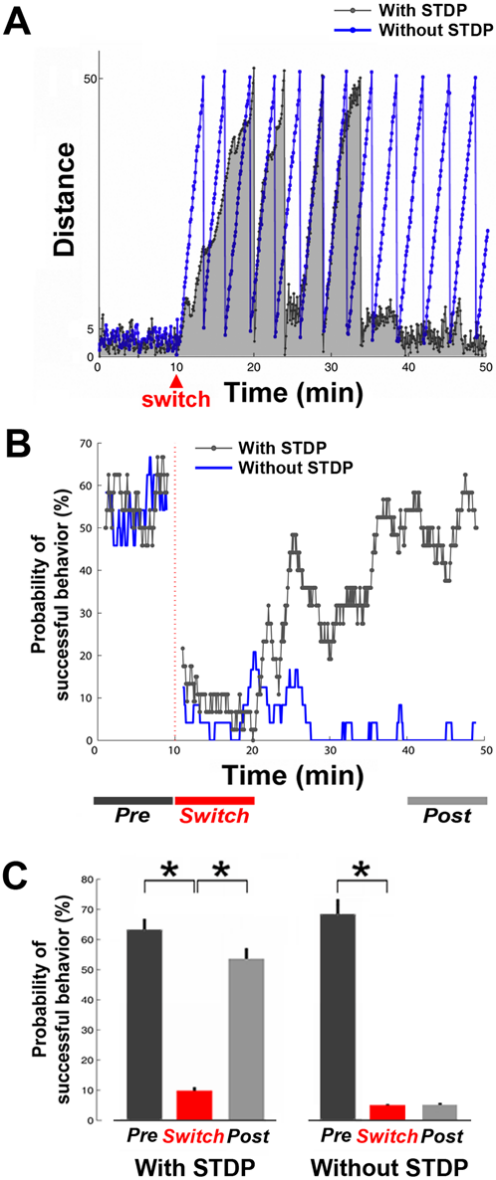
Network plasticity was essential for successful learning in the system. The successful adaptation in the overall system was contributed by learning in the network, and was not solely a product of the adaptive process in the artificial training algorithm. (A) The distances between the animat and the origin in a successful-learning simulation (with STDP, gray curve with gray shading for clarity) and the corresponding simulation without STDP (blue curve). The desired behavior could not be restored without the STDP algorithm. (B) The comparison of learning curves, defined as the change in probability of successful behavior over time, for simulations in (A). (C) Among 10 original successful-learning simulations, the average probability of successful behavior before the switch was 63.3±3.5%, dropped significantly to 9.8±1.1% after the switch (*p<5e-4, Wilcoxon signed-rank test), and increased significantly back to 53.6±3.5% when the desired behavior was restored (*, p<5e-4). These periods are shown in (B) (*Pre*: the 10 minutes before the switch; *Switch*: the 10 minutes immediately after the switch; and *Post*: the last 10 minutes). The probabilities of successful behavior in *Pre* and *Post* were comparable (p = 0.09). For all corresponding simulations without the STDP algorithm, the probability of successful behavior before the switch was 68.4±4.6% (n = 10 simulations without STDP), dropped significantly to 6.2±0.8% after the switch (*p<5e-4), but showed non-significant increase by the last 10 minutes of the simulation (6.4±0.9%; p = 0.91). This indicates that network long-term plasticity was essential for successful learning in the closed-loop system.

In order to verify that the successful adaptation in the overall system was contributed by learning in the network, and not solely by the adaptive process in the artificial training algorithm, we repeated the original successful-learning simulations with the STDP algorithm turned off. We found that the desired behavior could not be restored without the STDP algorithm, or long-term plasticity, in the network. This also rules out frequency-dependent synaptic depression as the adaptation mechanism, since that algorithm was left turned on. The comparison of the animat's movement in one successful-learning simulation and its corresponding simulation without STDP is shown in Figure 7, and the comparison of learning curves is shown in Figure 7B.

Among all original successful-learning simulations, the average probability of successful behavior before the switch was 63.3±3.5% (n = 10 successful-learning simulations), dropped significantly to 9.8±1.1% after the switch (p<5e-4, Wilcoxon signed-rank test), and increased significantly back to 53.6±3.5% after 88.6±12.2 minutes when the desired behavior was restored (p<5e-4) (Figure 7C). The probability of successful behavior after the switch was comparable to that before the switch (p = 0.09). For all corresponding simulation without STDP algorithm, the probability of successful behavior before the switch was 68.4±4.6% (n = 10 simulations without STDP), dropped significantly to 6.2±0.8% after the switch (p<5e-4), but showed no significant increase at the end of the simulation (6.4±0.9%) (p = 0.91) (Figure 7C). This indicated that network long-term plasticity was essential for successful learning in the closed-loop system.

### Experiment 5: Successful Learning Required Different PTSs at Different Times

**Figure 8:**
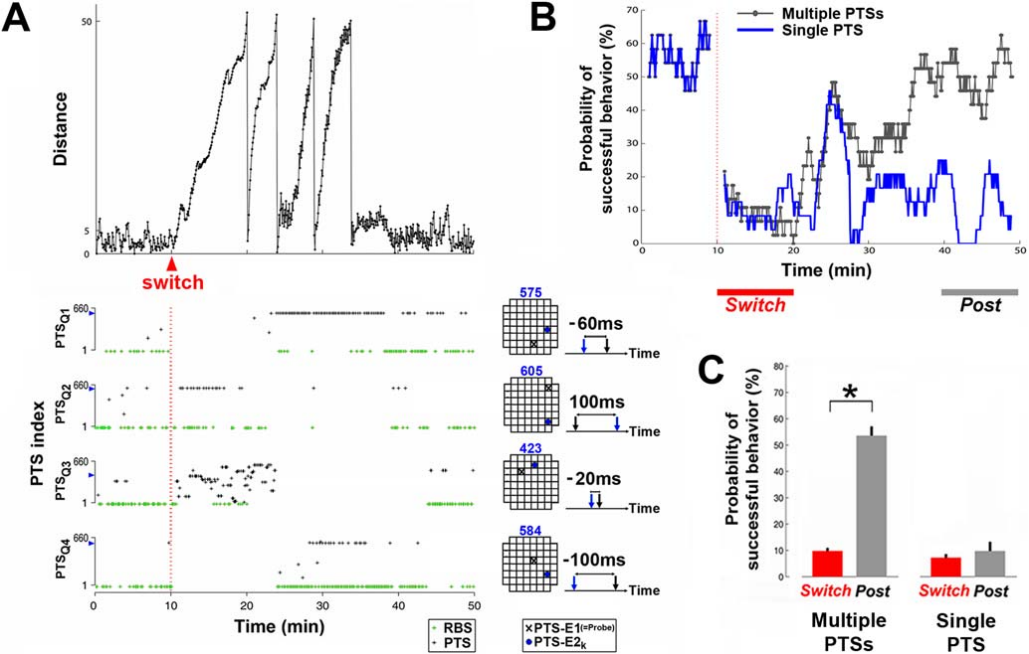
Successful adaptation required not only one PTS but a certain sequence of PTSs. (A) The training history of a successful-learning simulation (the distance measure is shown on the top panel). PTSs delivered from four different pools (PTS_Q1_–PTS_Q4_) are shown as black crosses, and the occurrences of RBS are shown as green crosses. From the 660 possible PTSs, the index of PTSs delivered most frequent in Q1, Q2, Q3, and Q4 were 575, 605, 423, and 584, respectively. The electrode locations and PTS_t_ of these four most frequent PTS patterns are shown on the right. For each pool, the location of the first electrode (PTS-E1, also the probe electrode, see Methods and Figure 1) is shown as a black X in the grids of 60 electrodes, and the second electrode (PTS-E2_k_) is shown as a blue dot. PTS_t_ between the PTS-E1 (black arrow) and PTS-E2_k_ (blue arrow) is also indicated for these four PTSs. (B) The learning curves of the successful-learning simulation shown in (A) (gray curve) and the corresponding simulation with only the most frequent PTSs available for training (blue curve, see Methods). In this example, the PTS patterns used for training in Q1, Q2, Q3, and Q4 were PTSs #575, 605, 423, and 584, respectively (see [A]). (C) The average probabilities of successful behavior during *Switch* and *Pre* periods (shown in [B]) in 10 original successful-learning simulations and 10 corresponding new simulations with only single PTS pattern available for training in each quadrant. For the original simulations, the average probability of successful behavior increased significantly back after the desired behavior was restored (*p<5e-4), while the average probability remained low for the simulations with single-PTS training (p = 0.61).

Different PTSs were delivered at different times before the desired behavior was restored. The training history from the same successful-learning example shown in Figure 7 is shown in Figure 8A. We hypothesized that the same PTS might have different effects at different points in time because the network would be in different states. Therefore, successful adaptations would require application of PTSs in a certain sequence. In order to test this hypothesis, we ran 10 additional simulations with only one PTS pattern available for training in each quadrant, instead of a pool of 660 PTSs as in the original stimulations (see Methods). These were the four most often used PTSs in the original simulations, one for each quadrant. For the example shown in Figure 8A, only PTS #575 was delivered in the new simulation when training was required due to unsuccessful movement in Q1.

We compared the original simulation and the corresponding new simulation by their learning curves (one example is shown in Figure 8B). The probability of successful behavior generally kept increasing after the switch for the original successful-learning simulation where multiple PTS patterns were available for training (gray curve), but not for the new simulation where only a single PTS pattern was available (blue curve).

A significant increase of the probability of successful behavior after the sensory mapping switch was found in the original successful-learning simulations (p<5e-4) (Figure 8D, and also Figure 7C). However, all 10 new simulations with only the four most frequent PTSs available showed no significant increase of the probability of successful behavior from immediately after the switch (9.2±1.0%) to the end of the simulation (10.1±3.7%) (p = 0.61, Wilcoxon signed-rank test) (Figure 8D). This shows that not only one PTS, but a *sequence* of different PTSs was needed in order to restore the desired behavior.

### Experiment 6: Training Contingent on Behavior Was Required for Successful Learning

We have demonstrated that successful adaptations to altered sensory mappings required a sequence of different PTSs, which was determined by the real-time feedback contingent on the animat's performance. In order to investigate the importance of behavior-contingent training for successful learning, we recorded the whole stimulation sequence (PTS and RBS) for each successfully adapted case and replayed it into the same network with the same initial state and same sensory-motor mapping. Different random seeds for fluctuations in neurons' membrane potentials and synaptic currents were used between the successful-learning simulations and the replayed training simulations. This difference would lead to different network responses, and thus different movement trajectories and different CPS sequences. However, the effect of non-training stimuli (CPSs and RBS) on shaping the network was insignificant, as shown in Figure 2. Therefore, whether the network could adapt to the new sensory mapping solely depended on the effect of training stimulation. The replayed training stimulation was no longer contingent on whether or not desired movement occurred.

**Figure 9:**
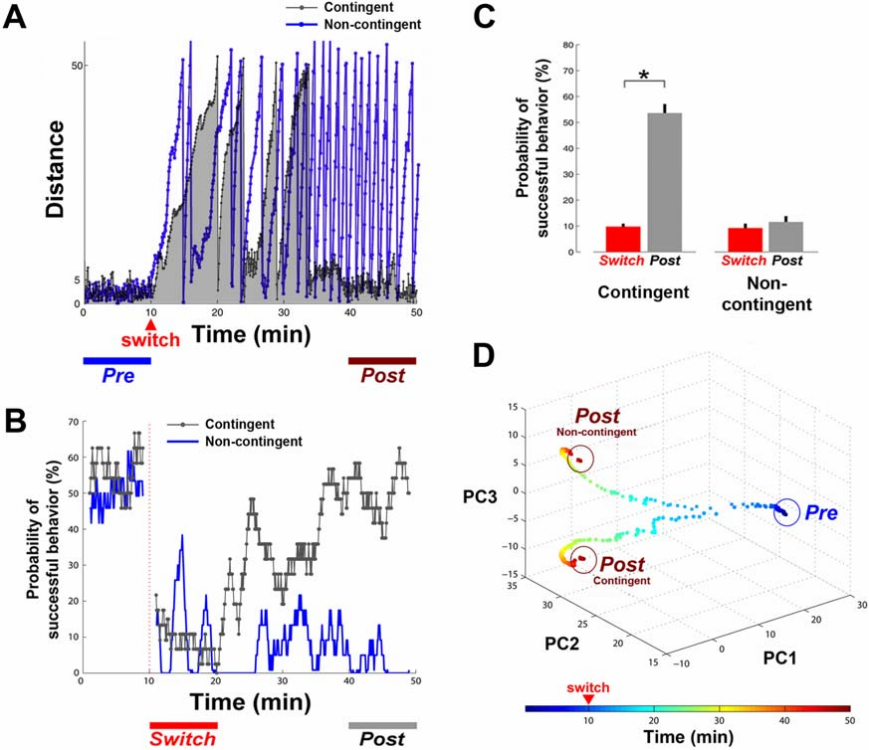
Behavior-contingent training was necessary for successful learning. A comparison between experiments with behavior-contingent training and with replayed training stimulation (non-contingent). (A) With real-time behavior-contingent training, the animat in this example was able to adapt to a sensory mapping switch and reach the desired behavior: moving in desired directions in each quadrant and staying within the inner circle (gray curve with gray shading for clarity). The adaptation was absent in the non-contingent experiment (blue curve). (B) The comparison of the learning curves corresponding for the example in (A). (C) The average probabilities of successful behavior in the 10 successful-learning experiments and the corresponding non-contingent experiments. With behavior-contingent training, the average probability of successful behavior in the last 10 minutes of the simulations (*Post* period shown in [B]) was significantly greater than that measured within 10 minutes after the switch (*Switch*) (*p<5e-4). In non-contingent experiments, the average probability of successful behavior in *Post* was comparable to that in *Switch* (p = 0.47). (D) The changes in all synaptic weights were visualized by Principal Components Analysis (PCA). The first three components (PC1 to PC3) of the network synaptic weights in the same example as (A) and (B) are plotted over time. Starting from the same initial synaptic weights, the network diverged to different synaptic weight distributions as the training became progressively less contingent on the network activity and the animat's performance. The circled periods, *Pre* and *Post*, are indicated at the bottom of (A).

In 10 stimulation-replay experiments, the animat was unable to show successful adaptation to the sensory mapping switch (shown as “non-contingent” in the example of Figure 9A), which had been successful with behavior-contingent training (shown as “contingent”).

A comparison of the learning curves for this example is shown in Figure 9B. With contingent training, a significant increase of the probability of successful behavior after the sensory mapping switch was found (p<5e-4) (Figure 9C, and also Figure 7C). However, with replayed training stimulation, the average probability of successful behavior in the last 10 minutes of the simulations was 11.6±2.2%, which is comparable to 9.2±1.8% measured within 10 minutes after the switch (p = 0.47) (Figure 9C).

In order to understand how successful (closed-loop) and replayed (open-loop) training stimulation shaped the network differently, we visualized the changes in weights of all synapses by using Principal Components Analysis (PCA). The first three components (PC1 to PC3) of the network synaptic weights for the contingent training simulation and the non-contingent training simulation example shown in Figure 9A are plotted over time (Figure 9D). Starting from the same initial synaptic weights, the network diverged to different synaptic weights distributions as the training became progressively less contingent on the network activity and the animat's performance.

### Experiment 7: The “Solution” for Successful Goal-Directed Behavior Is Not Unique

**Figure 10:**
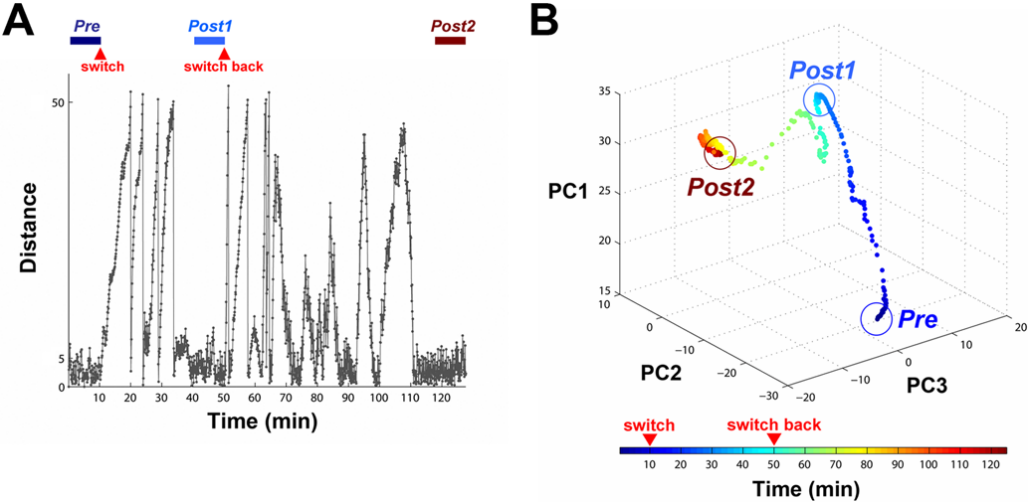
The “solution” for successful goal-directed behavior is not unique. The network re-adapted to reapplication of the original sensory mapping via a different state of network synaptic weights. (A) After the network adapted to a switch of the sensory mapping (*Post1* period), the sensory mapping was switched back to see whether the network could re-adapt to the original sensory mapping. One example is shown. The animat was able to restore the desired behavior (*Post2*) after the switch-back. (B) After adaptation to the switch-back, the animat showed the same desired behavior under the same sensory mapping, but with a different set of network synaptic weights. Multiple solutions existed for the desired behavior.

We have demonstrated that two different sets of network synaptic weights that were responsible for the desired behavior under two different sensory mappings (*Pre* and *Post-contingent* in Figure 9D). We then further investigated whether under a specific sensory mapping, the desired behavior could only be exhibited by a specific set of network synaptic weights. After the network adapted to the switched sensory mapping, we switched the sensory mapping back to the original sensory mapping to see whether the network could re-adapt to the original mapping (Figure 10). After the switch-back, the behavior-contingent patterned training stimulation was able to restore the desired behavior under the original sensory mapping (Figure 10A), but with a different set of network synaptic weights (Figure 10B). This indicates that multiple synaptic configurations, or “solutions”, existed for the desired behavior.

## Discussion

We demonstrated that an embodied simulated network could be shaped by patterned training stimulation into desirable states capable of expressing meaningful behavior. We applied a switching of the sensory mapping and measured the network's ability to rewire itself in order to restore the desired behavior under a new mapping. Previous studies have shown that functional visual projections routed into non-visual structures can change the modality of the cortex [29],[30]. This rewiring process was also found to restore function in the olfactory bulb following injury or neurological disease [31]. Successful rewiring observed in the random network suggests that cultured networks could be a useful model to investigate functional reorganization in cortical circuits after deafferentation or changes in sensory contingencies.

We exploited structured stimuli and detailed activity metrics [16] incorporating spatial information to show that with training contingent on the animat's behavior, the network was capable of learning associations between multiple sensory inputs and motor outputs (Experiment 2). We further showed that successful learning required proper selection of stimuli to encode sensory inputs (Experiment 3), and a variety of training stimuli (Experiment 5) with adaptive selection contingent on the animat's behavior (Experiment 6). We also found that the solution for a desired behavior was not unique (Experiment 7) and could be achieved through different paths of training. These results shed light on the complexity and flexibility of the learning process in neural networks.

### Effects of RBS in Simulated and Living Cortical Networks

RBS was hypothesized to negate “attractors” in network synaptic weight distributions caused by spontaneous activity (mainly network-wide synchronized bursts of activity called barrages), and to prevent network synaptic weights from drifting to such attractors after inducing plasticity with electrical stimulation [19]. RBS with an aggregate frequency of 1 Hz reduced the occurrence of spontaneous barrages by at least 10 times in the simulated network and dissociated cortical cultures [19]. By reducing the occurrence of spontaneous barrages, the network synaptic weights were mainly affected by activity evoked by RBS. Since RBS was random spatially and temporally, the evoked activity had an unbiased randomizing effect on changing network synaptic weights. In a different approach, a barrage-control stimulation protocol consisting of a group of electrodes cyclically stimulated with an aggregated frequency of 50 Hz was found to completely eliminate spontaneous barrages [32]. Similar to RBS, the barrage-control stimulation stabilized tetanus-induced plasticity in dissociated cortical cultures (Madhavan R, Chao ZC, Potter SM, unpublished data). However, different mechanisms might be involved. RBS evoked network-wide responses with unbiased spatiotemporal structure, while the barrage-control stimulation desynchronized spontaneous activity into spatially localized and temporally dispersed responses.

In this study, the aggregate stimulation frequency of RBS was increased from 1 to 3 Hz so that the amount of stimulation in RBS and PTS were comparable. RBS did stabilize network synaptic weights (the network synaptic weights were clustered in *Pre* period in Figure 9D) and also stabilized the network input-output function (see Figure 2).

### Selection of Stimuli for Sensory Encoding

Even though sharing the same network connectivity and the same PTS pools, some simulations showed successful learning and others were unsuccessful. Therefore, we concluded that the selection of CPSs for sensory encoding, which was the only remaining difference, was crucial for determining the success of adaptation. We found that the stimulations used to encode sensory inputs should evoke neither overly localized nor largely overlapped responses. Too much localization reduced the possibility to improve movement directions in switched quadrants, and too much overlap caused unwanted changes in un-switched quadrants. These results suggest a certain level of independence is required between responses to stimulations used to encode different sensory inputs, which could be achieved by using smaller and distinct recording areas to determine movement, or by offsetting the CA through the motor mapping transformation so that the probability of a CA to point in different directions is more uniform. Furthermore, correlated changes in responses to different sensory inputs could also be avoided by using training stimulation that only causes localized plastic changes. These findings could instruct the designs of implant electrode geometries and feedback stimulation patterns in prosthetics to achieve a more efficient and effective adaptation.

### Long-Term Plasticity and Successful Adaptation

We showed that long-term plasticity in the network (STDP) was essential for the adaptation in the overall system (see Figure 7). Short-term plasticity (frequency-dependent synaptic depression, see Methods and Supplemental Material Text S1) alone was not able to achieve successful adaptation (Figure 7). Furthermore, learning curves indicate that fewer training stimuli were required to maintain the desired behavior after the system had adapted (see Figure 7B and Figure 8B). These suggest that the improved performance was not due to short-term elastic responses to the stimulation. Elastic change was observed in dissociated cultures where the neurons' responsiveness adapted to very low frequency stimulation but relaxed back within minutes after stimulation was removed [33],[34].

### Different Training Schemes

Using paired pulses with different stimulation electrodes and different inter-pulse intervals was one possible design for training. More optimal training algorithms likely exist. Using stimulation sequences with more than two stimuli could help shape the network synaptic weights to a desired state, since they might evoke a greater variety of response patterns and produce different behaviors. However, the tradeoff is that a larger pool of possible training stimuli could lead to a longer training duration before successful adaptation. Furthermore, a different algorithm to adaptively update the probability of selecting PTSs might better find appropriate PTSs and remove unhelpful ones in the pool.

The simulated network was used to explore many different possible sensory-motor mappings and training algorithms (not described here) because of savings in preparation time and an ability to monitor all synaptic weights. The described algorithm successfully demonstrated adaptive goal-directed behavior with multiple sensory-motor mappings. This closed-loop algorithm is not restricted to a particular type or a particular number of sensory-motor mappings. Integrate-and-fire networks have been used previously for demonstrating goal-directed learning [35],[36]. In this work, we constructed a simulated network, specifically to mimic living MEA cultures, in order to find a closed-loop design that might be applicable to show goal-directed learning living cultures. In another study, we tested our closed-loop algorithm in a cortical network cultured over an MEA, where we successfully avoid Type I and Type II failure to train a living network to control the movement of an animat in a desired direction (Chao ZC, Bakkum DJ, Potter SM, unpublished data). Studying neural networks' basic computational properties, such as parallel signal processing and learning, by working with simulated/living *in vitro* networks could lead to direct development of more advanced artificial neural networks, more robust computing methods, and even the use of neurally controlled animats themselves as biologically-based control systems.

## Supporting Information

Text S1 (5.41 MB DOC)

Text S2 (0.24 MB DOC)

Movie S1 Movie of a successful-learning simulation. The trajectory, the trajectory around the inner circle (zoom-in), and the animat's distance from the origin in a successful-learning simulation are shown. A switching of the sensory mappings in Q1 and Q3 was applied after 10 minutes into the simulation. The animat's position is indicated as a blue dot. The trajectory in the zoom-in panel is indicated in different colors for different quadrants after the switch. The animat moved outward in Q1 and Q3 immediately after the switch, and restored the desired behavior after t = 20 min.
(6.78 MB MOV)
