# Supplementary material for: Shaping Embodied Neural Networks for Adaptive Goal-directed Behavior
Source: PLoS Comput Biol. 2008 Mar 28;4(3):e1000042. doi: 10.1371/journal.pcbi.1000042 (PMC2265558; doi:10.1371/journal.pcbi.1000042)
Supplement: Text S1 — (5.41 MB DOC) [file pcbi.1000042.s001.doc]

| **Supplemental Materials Text S1**  **Detailed features of the simulated network** |
| --- |

The exponential Euler method was used for numerical integration [1]. The values of parameters and their implementations in CSIM are described later in tables. Most of the parameters in the simulated network were approximated from studies of acute slices [2,3], retinal networks [4], and from the simulation of neocortical networks [5-7], others were determined in various validation experiments.

**Neuron model:**

1,000 neurons were placed randomly in a 3 mm by 3 mm area (Figure S1-1B). In real MEAs, we plate rat cortical neurons in an area larger than the dimension of the electrode grid, which is 1.4 mm by 1.4 mm (Figure S1-1A), to ensure every electrode has neurons around it. The 3 mm by 3 mm area for the simulated network is an estimation.

*Leaky integrate-and-fire (LIF) neuron:*

A standard LIF neuron model was implemented where the membrane potential *V_m_* of a neuron is given by:

$\tau_{m}\cdot\frac{dV_{m}}{dt}=-(V_{m}-V_{rest})+R_{m}\cdot(I_{syn}+I_{noise})$ [Equation S1-1]

*V_m_*: membrane potential

*R_m_*: membrane resistance

*τ_m_*: the membrane time constant, (= *C_m_***R_m_*), where *C_m_*: membrane capacitance

*I_syn_*: the current supplied by the synapses

*I_noise_*: a Gaussion random variable with zero mean and a given variance noise


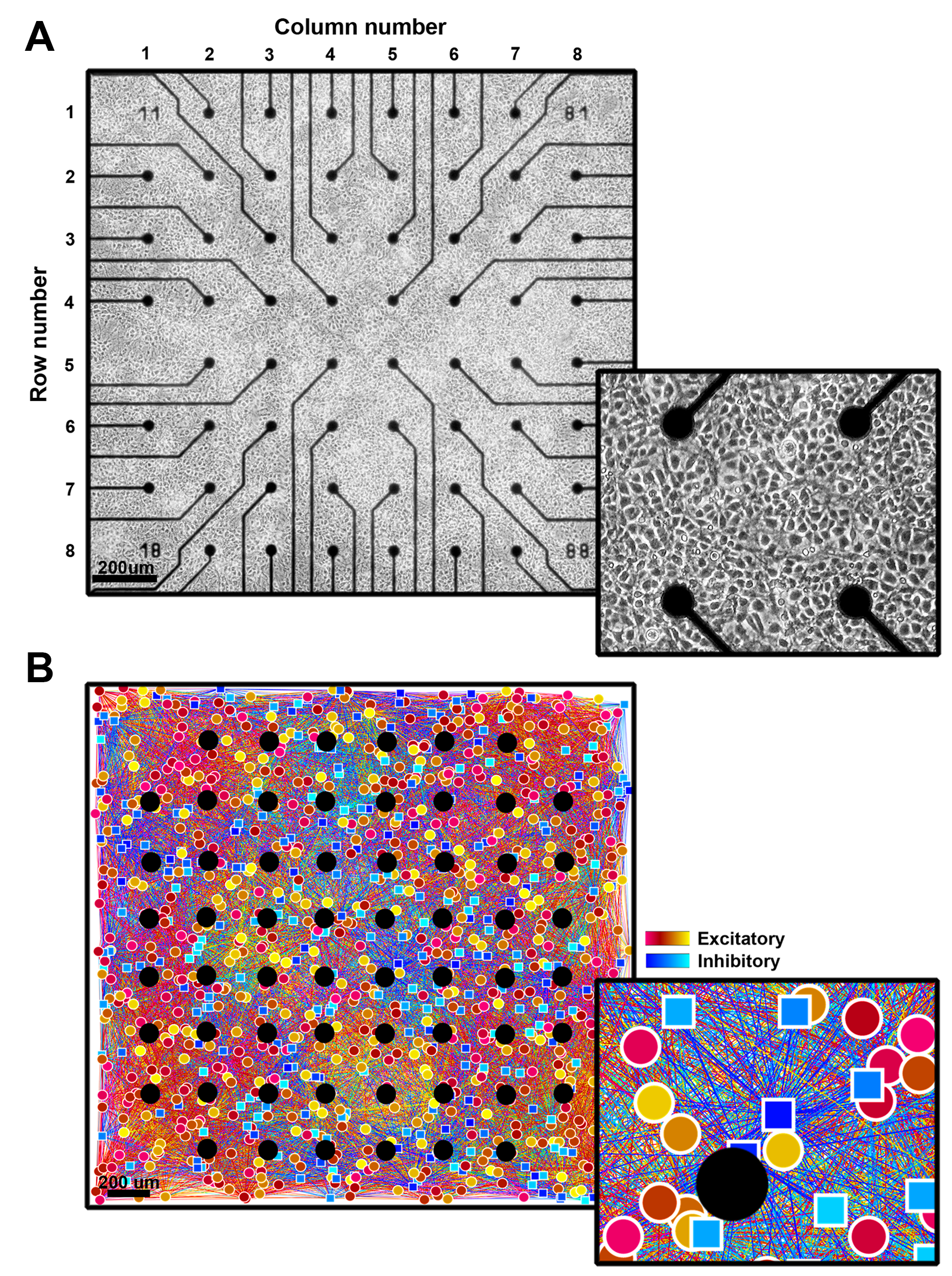


Figure S1-1. Living MEA culture vs. simulated network**:**

The simulated neural network and stimulation electrodes were constructed to mimic the dissociated cultured network and MEA setup. **A.** A view of a living MEA culture with 59 electrodes. **B.** The structure of a simulated network with 1,000 LIF neurons located in a region of 3 mm by 3 mm. The reddish circles indicate the excitatory neurons, the bluish squares indicate the inhibitory neurons, the reddish lines represent the excitatory synapses, and the bluish lines represent the inhibitory synapses (see colorbars). All neurons and synapses are shown. The locations of electrodes are shown in black circles.

At time *t*= 0, *V_m_* is set to *V_init_*. If *V_m_* exceeds the threshold voltage *V_thresh_*, it is reset to *V_reset_* and hold there for the length *T_refract_* of the absolute refractory period. An example of the postsynaptic current and the membrane potential of a neuron receiving several action potentials is shown in Figure S1-1.


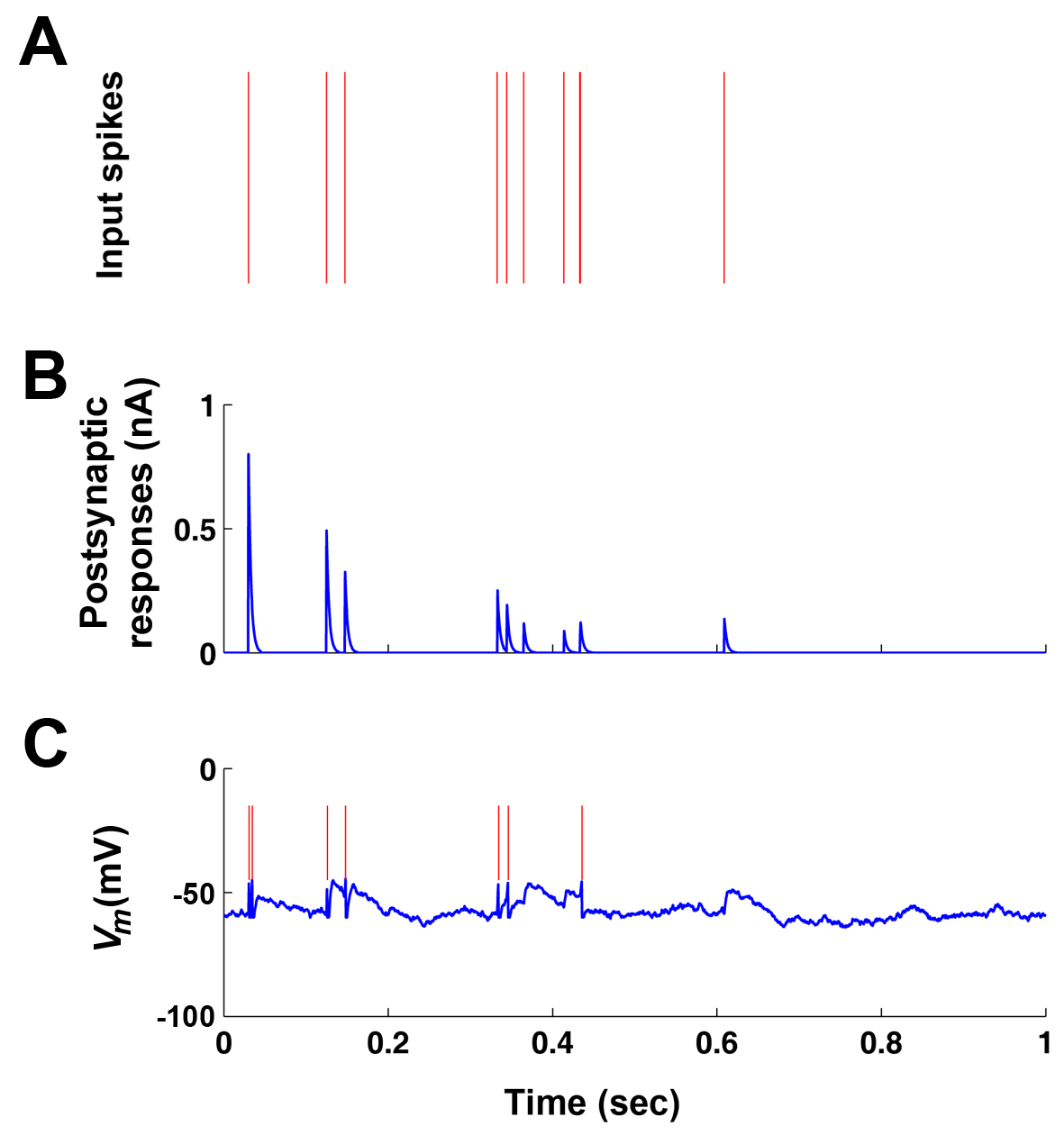


Figure S1-2. Evoked spikes in a LIF neuron**:**

Spikes from another neuron (**A**) evoked synaptic currents at the postsynaptic neuron (**B**), which increase the membrane potential (*V_m_*) in the postsynaptic neuron. A spike (red vertical lines) is evoked at the neuron, when *V_m_* is higher than a threshold (**C**).

*Self-firing:*

30% of the neurons (self-firing neurons) had *I_noise_* (see Equation S1-1) with variance at a high enough level to initiate spikes [8,9], while the rest exhibited only subthreshold fluctuations. Different levels of the standard deviation of *I_noise_* introduced to the self-firing neurons caused different spontaneous activity patterns: the rate of synchronized spontaneous bursting increased as the standard deviation of *I_noise_* increased (Figure S1-3). The bursting rate found in living cortical cultures is generally between 1 to 10 bursts per minute. In order to obtain the similar result, the standard deviation of 30 nA was added at each integration time step for self-firing neurons, and 10 nA was added for non-self-firing neurons according to the results shown in Figure S1-3.


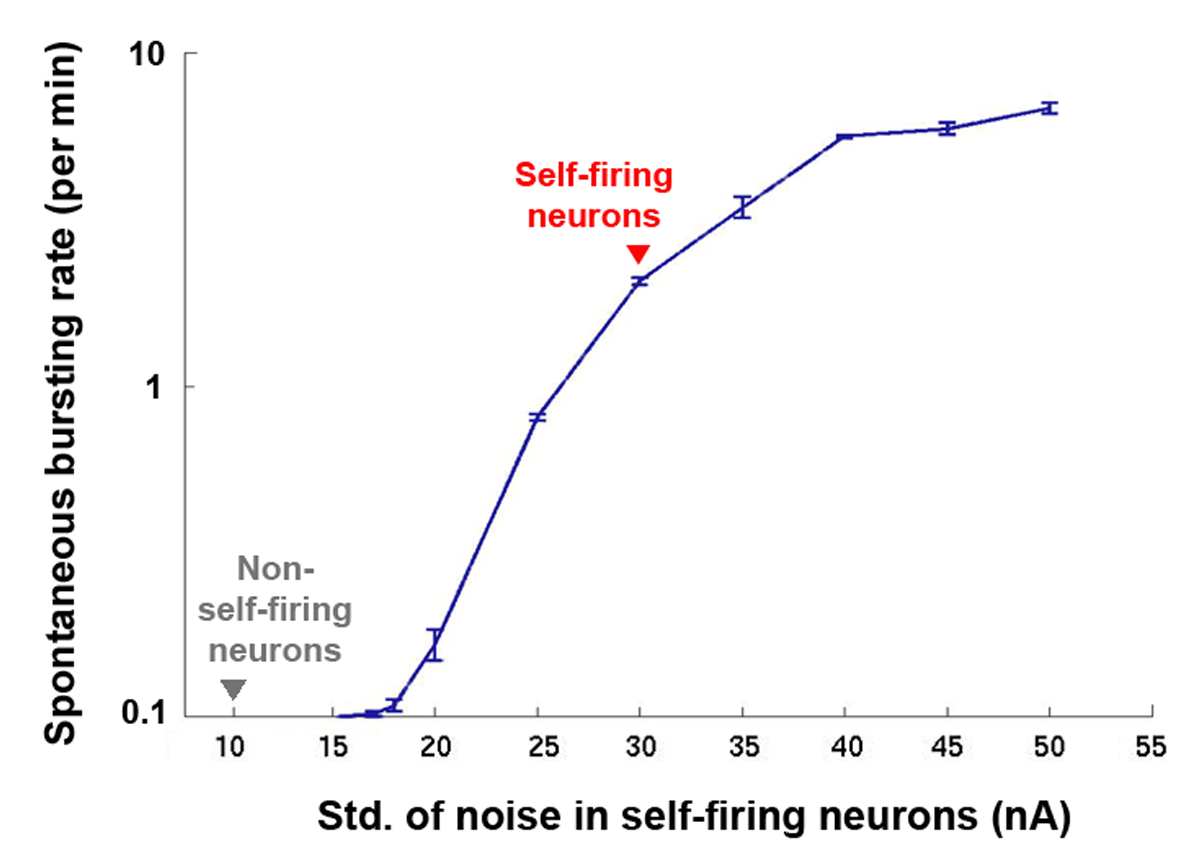


Figure S1-3. Determination of noise levels in self-firing and non-self-firing neurons**:**

The spontaneous bursting frequency increases as the standard deviation of the Gaussian noise added at each integration time step increases. For the non-self-firing neurons, the noise level was set at 10 nA, where the neurons only exhibited subthreshold fluctuation without spontaneous firing. For the self-firing neurons, the noise level was set at 30 nA, where spontaneous network bursting occurred at 1 to 10 bursts per minute, which is similar to the frequency found in living MEA cultures.

**Synapse model:**

*Excitatory vs. inhibitory:*

70% of the neurons were excitatory, which connected to other postsynaptic neurons with excitatory synapses, and the other neurons were inhibitory (30%) [10]. The setup of excitatory and inhibitory synapses is described later. The relations between several properties of spontaneous bursting and the proportion of inhibitory neurons are shown in Figure S1-4. We observed at most 15% GABAergic neurons in our cultures (unpublished data), which were within the 0- 30% range where the burst frequency, which is reversely proportional to the inter-burst interval (Figure S1-4A), and the burst duration (Figure S1-4B) remained at constant levels.


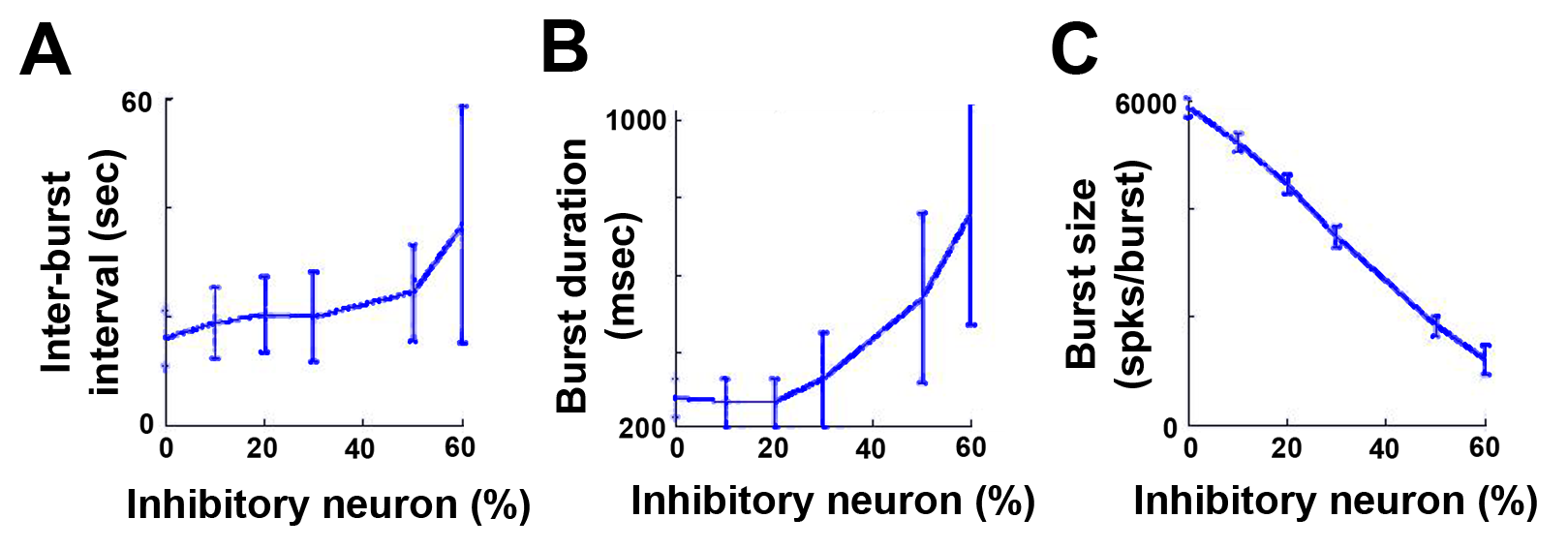


Figure S1-4. The size of inhibitory neuron population affected spontaneous bursting properties**:**

Spontaneous bursts were observed in the simulated network even without inhibitory neurons (100% excitatory neurons). The proportion of inhibitory neurons affected the interval between two consecutive bursts (**A**), the duration of a burst (**B**), and the number of spikes within a burst (**C**).

*Frequency-dependent dynamics:*

All synapses were frequency-dependent [3,5] to model synaptic depression; the synaptic efficacy was determined by the probability of release of neurotransmitters depending on the mechanism of frequency dependence [11,12]. The model is based on earlier concepts of the refractoriness of the release process [13], which can be rephrased by stating that the fraction (*U*) of the synaptic efficacy used by an action potential (AP) becomes instantaneously unavailable for subsequent use and recovers with a time constant of *D*. The fraction of available synaptic efficacy is termed *R*. The running value of *U* is referred to as *u*, where *U* remains a parameter that applies to the first AP in a train. *u* decays with a single exponential, *τ*, to its resting value *U*. The frequency-dependent dynamics are governed by the following equations:

$\begin{matrix} R_{1}=1-U \\ R_{n+1}=R_{n}\cdot(1-u_{n+1})\cdot\exp\left(\frac{-isi}{D}\right)+1-\exp\left(\frac{-isi}{D}\right) \\ u_{n+1}=u_{n}\cdot\exp\left(\frac{-isi}{\tau}\right)+U\cdot\left(1-u_{n}\cdot\exp\left(\frac{-isi}{\tau}\right)\right) \\ EPSP_{n}=W\cdot\exp\left(\frac{-\Delta t}{\tau}\right)\cdot R_{n}\cdot U \end{matrix}$ [Equations S1-2]

*R_n_*: the fraction of available synaptic efficacy at time step *n*

*U*: utilization of synaptic efficacy

*u_n_*: and the running value of *U* at time step *n*

*isi*: the time between *nth* and *(n+1)th* APs

*D*: time constant of recovery from the depression

*EPSP_n_*: excitatory postsynaptic potential and time step *n*

*W*: the synaptic weight

*Δt*: the time from previous arrival of AP to the simulation time

*τ*: the time constant of decay of responses

Intuitively, the excitatory postsynaptic potential (EPSP) of the synapse is proportional to the synaptic weight (*W*), the available synaptic efficacy (*R_n_*), and the initial capacity (*U*); and is increased when a presynaptic spike hits the synapse, otherwise decays exponentially with the time constant *τ*.

*Spike-timing-dependent plasticity (STDP):*

Both long-term potentiation (LTP) and long-term depression (LTD) of synapses are induced through STDP: firing of a postsynaptic neuron immediately after a presynaptic neuron results in LTP of synaptic transmission, and the reverse order of firing results in LTD [14-17]. Here, several advanced features were incorporated in the basic model (see below). The resulting STDP rule that governed the modification of synaptic weights between two monosynaptically connected neurons is shown in Figure S1-5.

*(1) Basic model:*

The basic STDP rule describes how a synaptic weight is modified by the timing between pre- and postsynaptic APs. In modeling studies, the basic rule is described as [2]:

$dw=\left\{\begin{matrix} W\cdot A_{+}\cdot\exp(\Delta t/\tau_{+}) \\ W\cdot A_{-}\cdot\exp(-\Delta t/\tau_{-}) \end{matrix}\right.\begin{matrix} if\begin{matrix} \Delta t<0 & \end{matrix} \\ if\begin{matrix} \Delta t\geq0 & \end{matrix} \end{matrix}$ [Equation S1-3]

*dw*: the normalized change of the synaptic weight (= *dW*/*W*)

*A_+_ / A-*: the maximum amounts of synaptic modification, which occur when *Δt* is

close to zero (both> 0)

*Δt*: pre-to-postsynaptic inter-spike intervals

*τ_+_ / τ-*: time constants of exponential decay of positive/negative learning window

Intuitively, presynaptic APs that follow postsynaptic APs (*Δt* < 0) produce long-term weakening of synapses (*dw* < 0), and APs arriving at the synapse with reverse order (*Δt* > 0) produce the opposite effect (*dw* > 0). The largest changes in synaptic efficacy (*A_+_* or *A-*) occur when the time difference between pre- and postsynaptic APs is small, and there is a sharp transition from strengthening to weakening as this time difference passes through zero. The effects of weakening and strengthening decrease as the time difference increases by time constants *τ-* and *τ_+_*, respectively (see Figure S1-5).

*(2) Saturation model:*

In order to prevent synaptic weights increasing or decreasing to unrealistic values, an upper bound and a lower bound were set. The updating functions (the first term on the right-hand side of Equation S1-4) were added to attenuate synaptic changes as a synapse approaches the upper or lower boundary of the allowed range.

$dw=\left\{\begin{matrix} (W_{\max}-W)^{\mu_{+}}\cdot A_{+}\cdot\exp(\Delta t/\tau_{+}) \\ (W-W_{\min})^{\mu_{-}}\cdot A_{-}\cdot\exp(-\Delta t/\tau_{-}) \end{matrix}\right.\begin{matrix} if\begin{matrix} \Delta t<0 & \end{matrix} \\ if\begin{matrix} \Delta t\geq0 & \end{matrix} \end{matrix}$ [Equation S1-4]

*W_max_* / *W_min_*: the maximal/minimal weight of the synapse

*μ_+_* / *μ-*: the extended multiplicative positive/negative update [18]

If *μ_+_* and *μ-* equal 1, then the updating functions linearly attenuate positive and negative synaptic weights when approaching *W_max_* and *W_min_*, respectively; otherwise, faster or slower nonlinear attenuation occurs. This prevents synaptic efficacies from becoming unnaturally excitatory or inhibitory and removing the problems caused by artificially clipping synaptic weights at their maxima.

*(3) Suppression model:*

The context of spike trains also affects how STDP operates. In visual cortical slices, the contribution of each pre- or postsynaptic spike pair to synaptic modification depends not only on the interval between the pair, but also on the timing of preceding spikes [7]. That is, activity-induced synaptic modification depends not only on the relative spike timing between the neurons, but also on the spiking pattern within each neuron. The firing history of each neuron influences the efficacy (*e_pr_*_e_ / *e_post_*) of subsequent APs, and further affects the role of each AP plays in STDP. This phenomenon is modeled as follow:

$\begin{matrix} \left\{\begin{matrix} e_{pre}=1-\exp(-\Delta t_{pre}/\tau_{pre}) \\ e_{post}=1-\exp(-\Delta t_{post}/\tau_{post}) \end{matrix}\right. \\ \\ W_{n+1}=W_{n}\cdot(1+e_{pre}\cdot e_{post}\cdot dw) \end{matrix}$ [Equations S1-5]

*e_pr_*_e_ / *e_post_*: the efficacy of the presynaptic/postsynaptic AP

*Δt_pr_*_e_ / *Δt_post_*: the time intervals between APs for calculating STDP and their previous APs in presynaptic/postsynaptic neurons

*τ_pr_*_e_ / *τ_post_*: the suppression time constant for presynaptic/postsynaptic neurons [7]

The synaptic change (*dw*) is “suppressed” by the efficacy of corresponding pre- and postsynaptic APs, which depends only on the interval from the preceding AP in the same neuron.

*(4) Overall modified model:*

Combining the standard STDP model with saturation constraints and the AP efficacy, the STDP synapse model was modified and governed by the following equations:

$$dw_{n}=\left\{\begin{matrix} (W_{\max}-W_{n})^{\mu_{+}}\cdot A_{+}\cdot\exp(\Delta t/\tau_{+}) \\ (W_{n}-W_{\min})^{\mu_{-}}\cdot A_{-}\cdot\exp(-\Delta t/\tau_{-}) \end{matrix}\right.\begin{matrix} if\begin{matrix} \Delta t<0 & \end{matrix} \\ if\begin{matrix} \Delta t\geq0 & \end{matrix} \end{matrix}$$

$\left\{\begin{matrix} e_{pre}=1-\exp(-\Delta t_{pre}/\tau_{pre}) \\ e_{post}=1-\exp(-\Delta t_{post}/\tau_{post}) \end{matrix}\right.$ [Equations S1-6]

$$W_{n+1}=W_{n}\cdot(1+e_{pre}\cdot e_{post}\cdot dw_{n})$$

The relation between *dW*/*W* (= *dw*) and *Δt* is shown in Figure S1-5.


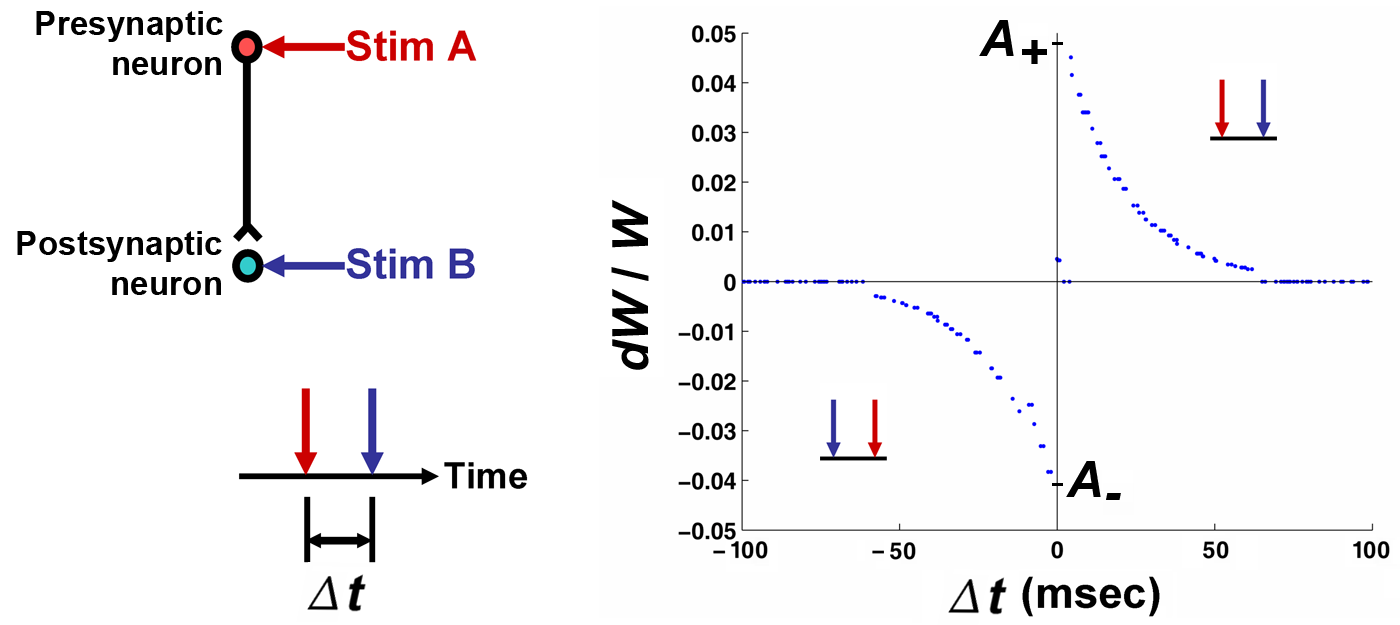


Figure S1-5. STDP synapse between two LIF neurons**:**

By varying the time interval (*Δt*) between spike inputs at two synaptically-connected neurons A and B, the normalized change in the synaptic weight of A to B followed the STDP rule. *A_+_* and *A-* represent the maximum amounts of synaptic potentiation and depression, respectively.

**Axon model:**

The axons in the simulated network connected neurons located at different locations, conducted APs traveling in the network with different propagation delays depending on the distance between neurons. For each axon, APs only travel one way. Two-way communications were allowed between two neurons using two separate axons, but no multiple axons connected one neuron to another in the same direction.

*Conduction velocity:*

The conduction delay was proportional to the distance between two connected neurons, and the conduction velocity was set to be 0.3 m/s [19].

*Connectivity:*

The distribution of the connection distances followed the distribution found by Segev and Ben-Jacob [20]: neurons tend to make many short connections but a few long ones as well (Figure S1-6). The number of connections per neuron followed a Gaussian distribution and each neuron had 50 ± 15 synapses onto other neurons.


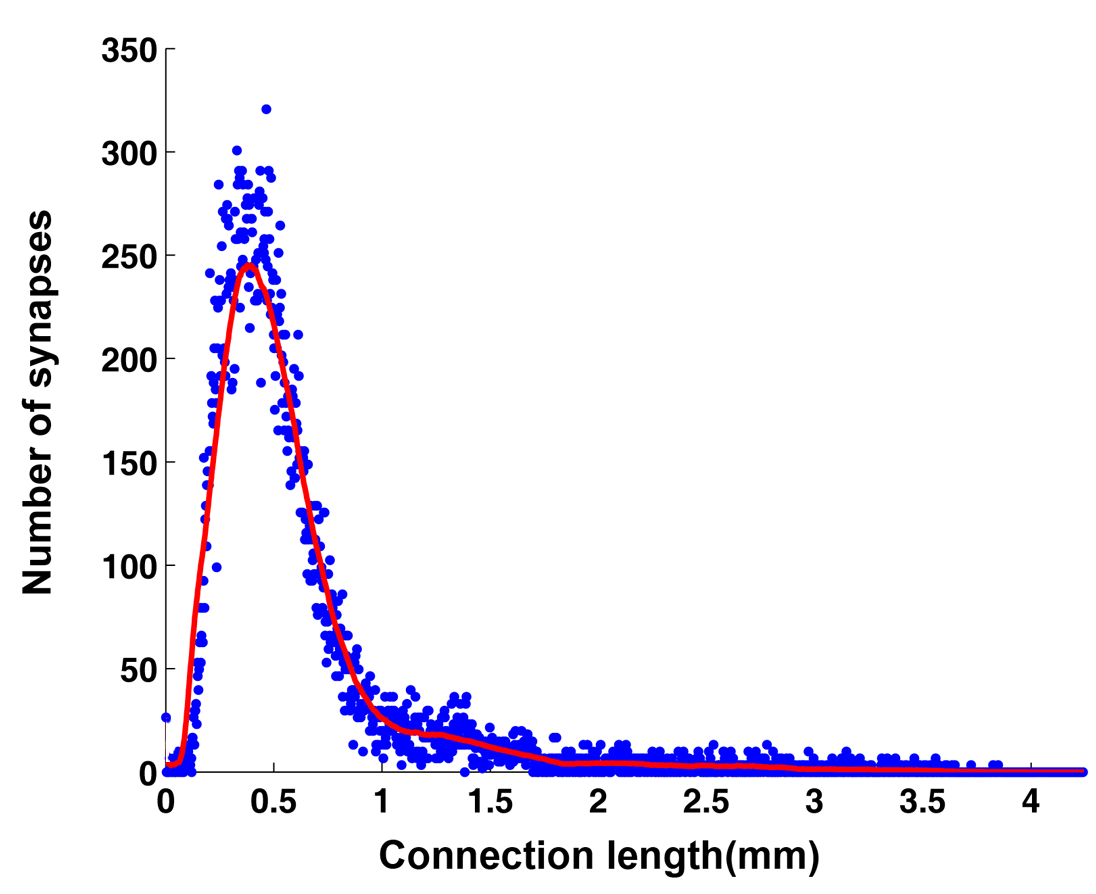


Figure S1-6. Distribution of synaptic connection lengths**:**

Neurons connected by axons tended to make many short connections but a few long ones as well. The longest connection was close to 4.24 mm, which is the length of the diagonal of the 3 mm by 3 mm area where the neurons were located.

**Electrode model:**

An 8 by 8 grid of electrodes with 333 µm inter-electrode spacing was included. The inter-electrode spacing, which was larger than the inter-electrode spacing of 200 µm in MEAs, was selected so that the distance from each peripheral electrode to the edge of the network were also the inter-electrode spacing. All electrodes could be used for stimulation, while 60 of these (except corner electrodes CR11, CR18, CR81 and CR88) were used for recording (Figure S1-1B). In real MEAs, electrode CR15 is the large ground electrode and is not used for recording/stimulation (not shown in Figure S1-1A). In the simulated network, each electrode recorded 5 ± 1 of the closest neurons, and stimulated 76 ± 12 of the closest neurons. These parameters were tweaked and optimized in various validation experiments (data not shown), since little experimental evidence exists for the number of neurons recorded or the range that one stimulus electrode could affect in cultured living networks.

**Simulation setup:**

All excitatory synaptic weights were initially set to 0.05 and could vary between zero and 0.1 due to STDP. At the maximal weight, each spike would have a 50% probability of evoking a spike in the postsynaptic neuron, due to its summation with intrinsic noise (Figure S1-7). The synaptic weights for the inhibitory connections were fixed at -0.05. The networks were run for 2 hours in simulated time until the synaptic weights reached the steady state, which took 3 to 4 hours in compute time (MATLAB 7.0, AMD Athlon processor, 2.08 GHz, 512 MB RAM). Most of the excitatory synaptic weights (93 ± 2%) were less than 0.01 or greater than 0.09 (Figure S1-8). This bimodal steady-state distribution of weights arose from the STDP rule, as previously observed by Song et al. [2], and Izhikevich and Desai [21]. The set of synaptic weights after 2 hours, which stabilized without external stimuli, was used for the subsequent simulation experiments as the initial state.


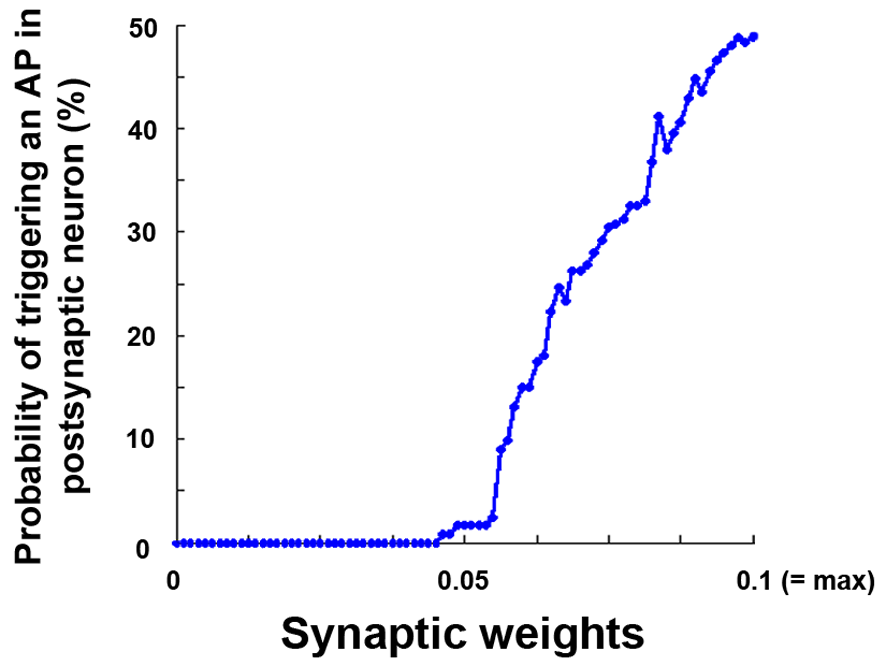


Figure S1-7. Relation between the synaptic weight and the firing probability of the postsynaptic neuron**:**

By varying the synaptic weight between two neurons, the probability of triggering an AP in the postsynaptic neuron was measured. At the maximal weight (0.1), each presynaptic spike would have a 50% probability of evoking a spike in the postsynaptic neuron, due to fluctuations in the postsynaptic neuron’s membrane potential.


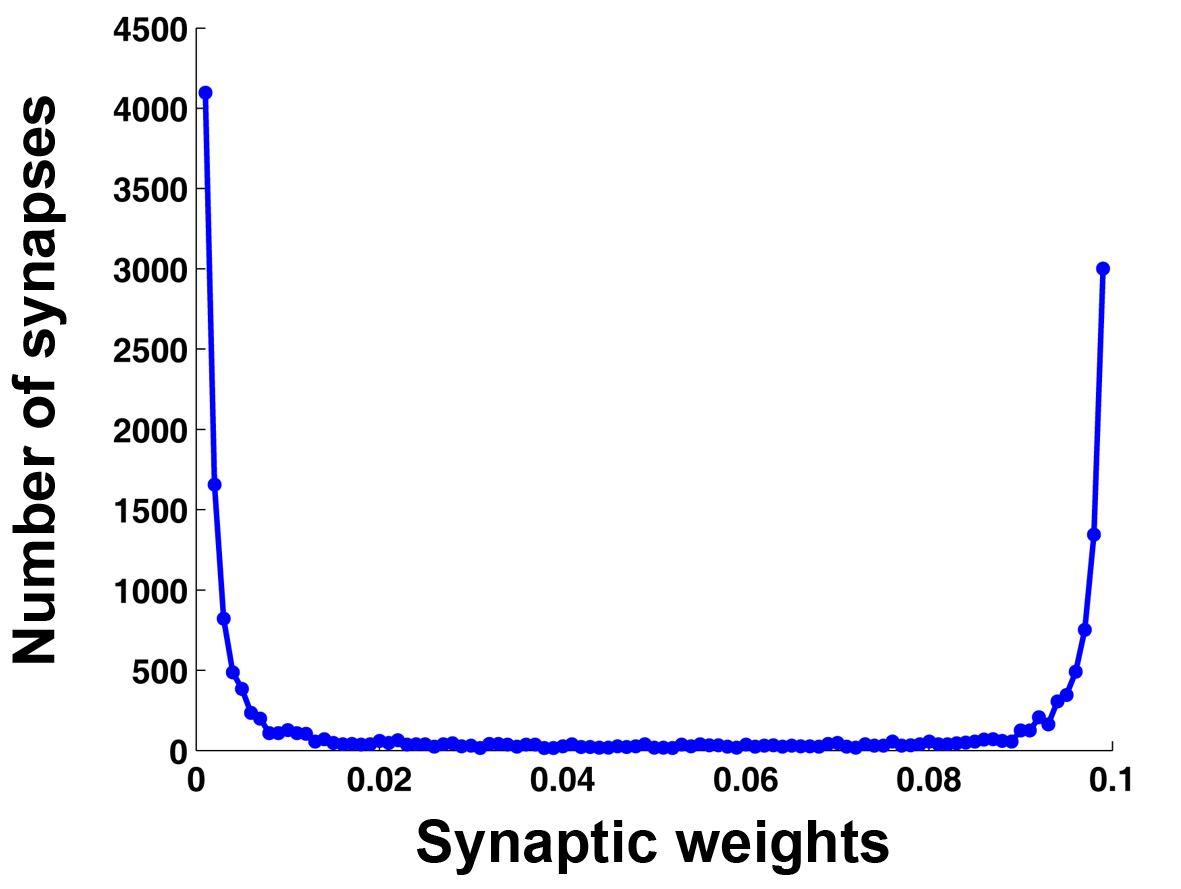


Figure S1-8. Bimodal distribution of network synaptic weights in equilibrium**:**

The network synaptic weights of excitatory synapses reached equilibrium with a bimodal distribution after 2 hours in simulation time. Most of the excitatory synaptic weights (93 ± 2%) were less than 0.01 or greater than 0.09.

**Simulation parameters:**

The parameters used in Neural Circuit SIMulator (CSIM) [22] and their implementations were shown below:

General simulation parameters:

| **Parameter** | **Value** | **in CSIM** |
| --- | --- | --- |
| Integration time step | 100 μsec | dt |
| Recording time step for APs | 100 μsec | MexRecorder:spikes:dt |
| Recording time step for synaptic weights | 10- 500 msec | MexRecorder:W:dt |

Network parameters:

| **Parameter** | **Value** | **Reference value** |
| --- | --- | --- |
| # of neurons | 1,000 | —— |
| # of synaptic connection | ~50,000 | —— |
| Synapses per neuron | 50 ± 15 | —— |
| % of inhibitory neurons | 30 | 20 [2]  25 [5] |
| % of self-firing neurons | 30 | ~30 [8,9] |

**Neuron parameters:**

— LIF neuron:

| **Parameter** | **Value** | **in CSIM** |
| --- | --- | --- |
| *V_resting_* | -70 mV | LifNeuron:Vresting |
| *V_init_* | -70 mV | LifNeuron:Vinit |
| *V_thresh_* | -54 mV | LifNeuron:Vthresh |
| *V_reset_* | -60 mV | LifNeuron:Vreset |
| *T_refract_* | 3 msec | LifNeuron:Trefract |
| *C_m_* | 3e-8 F | LifNeuron:Cm |
| *R_m_* | 1e6 ohm | LifNeuron:Rm |
| *τ_m_* | 30 msec | —— |

— Self-firing:

| **Parameter** | **Value** | **Reference value** | **in CSIM** |
| --- | --- | --- | --- |
| *I_noise_* (self-firing) | 30 nA | By optimization | LifNeuron:Inoise |
| *I_noise_* (non-self-firing) | 10 nA | By optimization | LifNeuron:Inoise |

**Synapse parameters:**

— General:

| **Parameter** | **Type** | **in CSIM** |
| --- | --- | --- |
| Type of excitatory synapses | STDP + frequency-dependent | DynamicStdpSynapse |
| Type of inhibitory synapses | Frequency-dependent | DynamicSpikingSynapse |

— Frequency-dependent (excitatory):

| **Parameter** | **Value** | **Reference value** | **in CSIM** |
| --- | --- | --- | --- |
| *R_0_* | 100% | —— | DynamicStdpSynapse:r0 |
| *U* | 0.5 | ~0.6 [23]  0.5 [5] | DynamicStdpSynapse:U |
| *u_0_* | 0.5 | —— | DynamicStdpSynapse:u0 |
| *D* | 800 msec | ~800 msec [23]  800 msec [5] | DynamicStdpSynapse:D |
| *F* | 1 sec | 1 sec [5] | DynamicStdpSynapse:F |
| *τ* | 3 msec | 5- 6 msec [5] | DynamicStdpSynapse:tau |

— Frequency-dependent (inhibitory):

| **Parameter** | **Value** | **Reference value** | **in CSIM** |
| --- | --- | --- | --- |
| *R_0_* | 100% | —— | DynamicSpikingSynapse:r0 |
| *U* | 0.5 | ~0.6 [23]  0.2 [5] | DynamicSpikingSynapse:U |
| *u_0_* | 0.5 | —— | DynamicSpikingSynapse:u0 |
| *D* | 800 msec | ~200 msec [23]  700 msec [5] | DynamicSpikingSynapse:D |
| *F* | 1 sec | 20 msec [5] | DynamicSpikingSynapse:F |
| *τ* | 3 msec | ~2 msec [24] | DynamicSpikingSynapse:tau |

— STDP (excitatory synapses only):

| **Parameter** | **Value** | **Reference value** | **in CSIM** |
| --- | --- | --- | --- |
| *A_+_* | 0.5 | 0.5% [2]  0.4% [5] | DynamicStdpSynapse:Apos |
| *A-* | 0.5*1.05% | 0.5*1.05% [5]  0.4% [5] | DynamicStdpSynapse:Aneg |
| *τ_+_* | 20 msec | 20 msec [5]  ~13 msec [7]  15 msec [5] | DynamicStdpSynapse:taupos |
| *τ-* | 20 msec | 20 msec [5]  ~34 nsec [7]  20 msec [5] | DynamicStdpSynapse:tauneg |
| *W_up_* | 0.1 | 0.5 [5] | DynamicStdpSynapse:Wex |
| *W_low_* | 0 | 0 [5] |  |
| *μ_+_* | 1 | —— | DynamicStdpSynapse:mupos |
| *μ-* | 1 | —— | DynamicStdpSynapse:muneg |
| *τ_pr_*_e_ | 34 msec | 28 msec [7] | DynamicStdpSynapse:tauspre |
| *τ_post_* | 75 msec | 88 msec [7] | DynamicStdpSynapse:tauspost |

**References:**

1. Hochbruck M, Lubich C, Selhofer H (1998) Exponential Integrators for Large Systems of Differential Equations. SIAM Journal on Scientific Computing 19: 1552-1574.

2. Song S, Miller KD, Abbott LF (2000) Competitive hebbian learning through spike-timing-dependent synaptic plasticity. Nature Neuroscience 3: 919-926.

3. Markram H, Gupta A, Uziel A, Wang Y, Tsodyks M (1998) Information processing with frequency-dependent synaptic connections. Neurobiology of Learning and Memory 70: 101-112.

4. Yang G, Masland RH (1992) Direct visualization of the dendritic and receptive fields of directionally selective retinal ganglion cells. Science 258: 1949-1952.

5. Izhikevich EM, Gally JA, Edelman GM (2004) Spike-Timing Dynamics of Neuronal Groups. Cerebral Cortex 14: 933-944.

6. Izhikevich EM (2005) Polychronization: Computation with Spikes. Neural Computation 18: 245-282.

7. Froemke RC, Dan Y (2002) Spike-timing-dependent synaptic modification induced by natural spike trains. Nature 416: 433-438.

8. Latham P, Richmond B, Nelson P, Nirenberg S (2000) Intrinsic dynamics in neuronal networks. I. Theory. J Neurophysiol 83: 808-827.

9. Latham PE, Richmond BJ, Nirenberg S, Nelson PG (2000) Intrinsic dynamics in neuronal networks. II. Experiment. Journal of Neurophysiology 83: 828-835.

10. Marom S, Shahaf G (2002) Development, learning and memory in large random networks of cortical neurons: Lessons beyond anatomy. Quarterly Reviews of Biophysics 35: 63-87.

11. del Castillo J, Katz B (1954) Statistical factors involved in neuromuscular facilitation and depression. The Journal of Physiology 124: 574-585.

12. Betz WJ (1970) Depression of transmitter release at the neuromuscular junction of the frog. The Journal of Physiology 206: 629-644.

13. Zucker RS (1989) Short-term plasticity. Annu Rev Neurosci 12: 13-31.

14. Desmond NL, Levy WB (1983) Synaptic correlates of associative potentiation/depression: an ultrastructural study in the hippocampus. Brain Res 265: 21-30.

15. Markram H, Lubke J, Frotscher M, Sakmann B (1997) Regulation of synaptic efficacy by coincidence of postsynaptic APs and EPSPs. Science 275: 213-215.

16. Bi G, Poo M (1998) Synaptic modifications in cultured hippocampal neurons: Dependence on spike timing, synaptic strength, and postsynaptic cell type. J Neurosci 18: 10464-10472.

17. Gerstner W, Kempter R, van Hemmen JL, Wagner H (1996) A neuronal learning rule for sub-millisecond temporal coding. Nature 383: 76-78.

18. Gutig R, Aharonov R, Rotter S, Sompolinsky H (2003) Learning Input Correlations through Nonlinear Temporally Asymmetric Hebbian Plasticity. Journal of Neuroscience 23: 3697-3714.

19. Kawaguchi H, Fukunishi K (1998) Dendrite classification in rat hippocampal neurons according to signal propagation properties - Observation by multichannel optical recording in cultured neuronal networks. Experimental Brain Research 122: 378-392.

20. Segev R, Ben-Jacob E (2000) Generic Modeling of Chemotactic Based Self-Wiring of Neural Networks. Neural Networks 13: 185-199.

21. Izhikevich EM, Desai NS (2003) Relating STDP to BCM. Neural Computation 15: 1511-1523.

22. Natschlager T, Markram H, Maass W (2003) Computer models and analysis tools for neural microcircuits. In: Kotter R, editor. Neuroscience Databases A Practical Guide. Boston: Kluwer Academic Publishers. pp. 123-138.

23. Markram H, Wang Y, Tsodyks M (1998) Differential signaling via the same axon of neocortical pyramidal neurons. National Acad Sciences. pp. 5323-5328.

24. Abbott LF, Varela JA, Sen K, Nelson SB (1997) Synaptic Depression and Cortical Gain Control. Science 275: 221-224.
