# Supplementary material for: Shaping Embodied Neural Networks for Adaptive Goal-directed Behavior
Source: PLoS Comput Biol. 2008 Mar 28;4(3):e1000042. doi: 10.1371/journal.pcbi.1000042 (PMC2265558; doi:10.1371/journal.pcbi.1000042)
Supplement: Text S2 — (0.24 MB DOC) [file pcbi.1000042.s002.doc]

| **Supplemental Materials Text S2**  **Context-control probing sequence (CPS) minimized the variability in probe responses** |
| --- |

The evoked responses to the last stimulus (probe) in CPSs were used to generate motor commands for the animat. We found that by controlling the stimulation context (the stimuli before the probe) with inter-pulse interval (IPI) between 200 to 400 msec, the variability in the probe responses could be minimized in both simulated networks and living MEA cultures (Figure S2-1).

In 5 stimulated networks and a living MEA culture, we delivered pairs of stimuli at two randomly selected electrodes. The stimuli at the first electrode (context) served as the context of the stimuli at the second electrode (probe). IPIs (time intervals from the context stimuli to the corresponding probe stimuli) were randomized between 100 msec and 2 seconds, and the time intervals between the probe stimuli and the next context stimuli were fixed at 2 seconds. By binning the IPIs with 100 msec moving window and 10 msec step, we grouped the IPIs into 181 groups. The variability of CAs of the probe responses, whose corresponding IPIs belonged to the same group, was calculated. The variability was defined as:

$Variability=\sqrt{\mathrm{var}\left(CA_{X}\right)+\mathrm{var}\left(CA_{Y}\right)}$ [Equation S2-1]

For each experiment, the variability calculated from different IPI bins was normalized by removing the mean then dividing by the standard deviation, which allowed the normalized variability to have negative values (see Figure S2-1). The mean ± SEM of the normalized variability from 20 experiments in 5 simulated networks (4 different stimulation pairs in each network) is shown in the left panel. The mean ± SEM of the normalized variability from 4 experiments in a living MEA culture (4 different stimulation pairs) is shown in the right panel. The relation between normalized variability and IPI was fitted with a 6 degree polynomial (red curves) and the 90% confidence intervals (red dash curves) were calculated. The minimum of the normalized variability was found between 200 to 400 msec in both simulated networks and the living MEA culture.

Compared to stimulating with random IPI, fixing IPI can reduce response variability. Using the same stimulation sequence, but instead of analyzing the responses per IPI, responses were analyzed for random selected IPIs. The average normalized variability from 1000 different random samplings is shown as the black horizontal lines. The results indicate that by controlling IPI and the context electrode, the variability in probe responses can be reduced with a minimum between 200 and 400 msec IPIs.

**
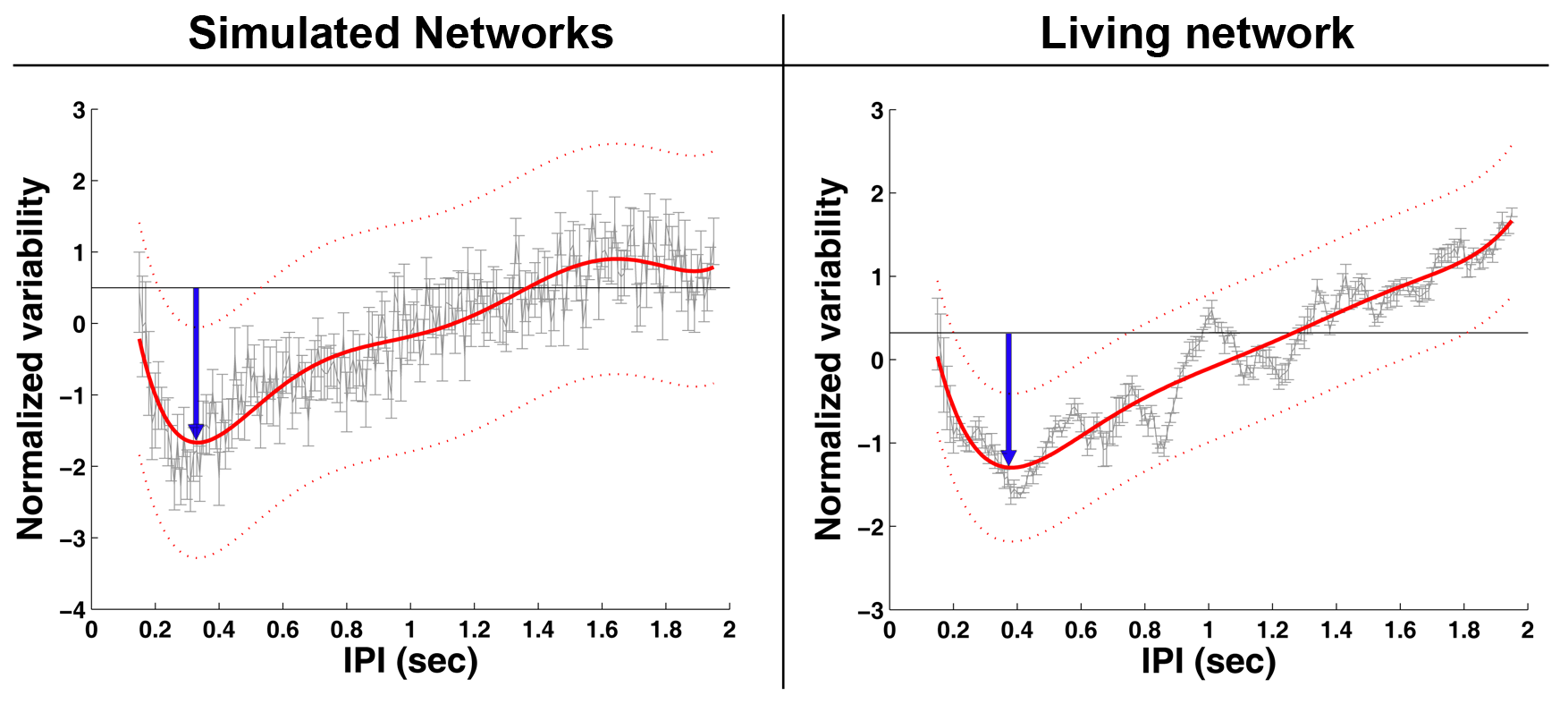
**

Figure S2-1. Relation between variability of probe responses and IPI in simulated and living networks**:**

The minimal variability in CAT of probe responses occurred with IPIs between 200 and 500 msec in both simulated networks (**Left**) and living MEA cultures (**Right**). Blue arrows indicate expected reduction in the variability of probe responses, when using optimal IPIs, compared to using randomly-timed stimuli preceding a probe.
